# The Evolution of Reproductive Isolation Beyond a Strong First Barrier in Speciation Between Micro‐Allopatric Host Races of a Phytophagous Ladybird Beetle, *Henosepilachna diekei*

**DOI:** 10.1002/ece3.74046

**Published:** 2026-07-17

**Authors:** Arif Maulana, Kei W. Matsubayashi, Naoyuki Fujiyama, Tri Atmowidi, Sih Kahono

**Affiliations:** ^1^ Department of Biology, Faculty of Mathematics and Natural Sciences IPB University (Bogor Agricultural University) Bogor West Java Indonesia; ^2^ Wellcome Sanger Institute, Tree of Life Programme Cambridge UK; ^3^ Darwin College University of Cambridge Cambridge UK; ^4^ Department of Environmental Sciences, College of Agriculture, Food and Environment Sciences Rakuno Gakuen University Ebetsu‐shi Hokkaido Japan; ^5^ Department of Biology, Faculty of Science Yamagata University Yamagata Yamagata Japan; ^6^ National Research and Innovation Agency Research Centre for Biota Systems, Kawasan Sains dan Teknologi Dr. (H.C.) Ir. H. Soekarno Bogor West Java Indonesia

**Keywords:** divergent selection, ecological speciation, host plant specialisation, host races, reduced egg hatchability, speciation continuum, kontinum spesiasi, penurunan daya tetas telur, ras inang, seleksi divergen, spesialisasi tumbuhan inang, spesiasi ekologi

## Abstract

Reproductive isolation often accumulates unevenly during speciation, with some barriers evolving early and others appearing only after further divergence. In host‐associated phytophagous insects, divergent host use can generate strong habitat isolation that evolves early in speciation and often results in sudden shifts towards strong reproductive isolation. However, whether additional sexual or postmating barriers can accumulate after this strong early ecological barrier has evolved remains unclear. In this study, we examined this question in the host‐specialised populations of the Southeast‐Asian herbivorous ladybird beetle, *Henosepilachna diekei*. We focused on the *Dicliptera–Mikania* race pair and compared it with the previously studied *Mikania–Leucas* race pair, representing two putative levels of host‐race differentiation. Combining laboratory crossing and field‐cage experiments, we investigated and quantified four potential isolating factors: premating isolation (habitat and sexual isolation), postmating‐prehatching isolation (reduced egg hatchability), and postmating‐posthatching isolation (F_1_ hybrid inviability). The *Dicliptera*–*Mikania* race pair showed larger genetic differentiation than the sympatric *Mikania*–*Leucas* race pair. Both race pairs showed strong habitat isolation, but only the *Dicliptera*–*Mikania* race pair showed additional later‐acting barriers, including reduced mating success, reduced egg hatchability, and reduced F_1_ hybrid survival. Our findings suggest that additional reproductive barriers can evolve even in the presence of exceptionally strong habitat isolation during host‐race divergence. Rather than replacing the importance of the initial host‐associated barrier, late‐acting barriers may accumulate and contribute to the stability of reproductive isolation. Consequently, the strength and dynamics of later‐acting barriers may depend on the evolutionary trajectory of each host‐race pair, reflecting diverse pathways through which speciation can unfold in host‐associated insects.

## Graphical abstract

Reproductive isolation often accumulates unevenly during speciation, but when an early ecological barrier such as host‐associated habitat isolation is already very strong, it remains unclear whether, and how rapidly, additional sexual or postmating barriers continue to evolve, or whether divergence is maintained primarily by this first barrier alone. Here, we examined this question in host‐specialised populations of the Southeast Asian herbivorous ladybird beetle *Henosepilachna* *diekei*, focusing on the *Dicliptera*–*Mikania* race pair and comparing it with the previously studied *Mikania*–*Leucas* race pair as two putative levels of host‐race differentiation. The presence of reduced mating success, reduced egg hatchability, and reduced F_1_ hybrid survival in the more genetically differentiated *Dicliptera*–*Mikania* pair suggests that sexual and postmating barriers may accumulate rapidly after strong host‐associated habitat isolation has evolved.

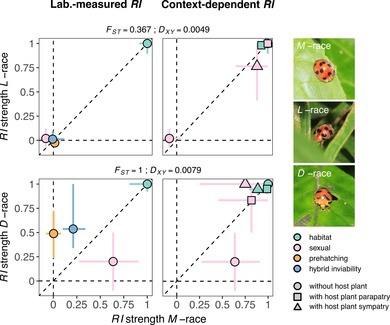

## Abstrak

Isolasi reproduktif umumnya terakumulasi secara tidak seragam dalam skala temporal selama proses spesiasi, dengan beberapa penghalang reproduktif berevolusi lebih awal, sementara yang lain baru muncul setelah divergensi lebih lanjut. Pada serangga fitofag yang berasosiasi dengan inang, penggunaan inang yang berbeda dapat menghasilkan isolasi habitat yang kuat, yang berevolusi pada fase awal spesiasi dan sering menyebabkan pergeseran level isolasi reproduktif yang mendadak. Namun, masih belum jelas apakah penghalang tambahan berupa isolasi seksual atau pascakawin dapat terus terakumulasi setelah penghalang ekologis awal yang kuat ini berevolusi. Dalam studi ini, kami mengkaji pertanyaan tersebut pada populasi terspesialisasi inang dari kumbang lembing herbivor Asia Tenggara, *Henosepilachna diekei*. Kami berfokus pada pasangan ras *Dicliptera*–*Mikania* dan membandingkannya dengan pasangan ras *Mikania*–*Leucas* yang telah dikaji sebelumnya, yang mewakili dua tingkat diferensiasi ras inang. Dengan menggabungkan percobaan persilangan di laboratorium dan percobaan kurungan lapangan, kami menyelidiki dan mengkuantifikasi empat faktor isolasi potensial: isolasi prakawin (isolasi habitat dan isolasi seksual), isolasi pascakawin‐prapenetasan (penurunan daya tetas telur), dan isolasi pascakawin‐pascapenetasan (ketidakviabilitas hibrida F_1_). Pasangan ras *Dicliptera*–*Mikania* menunjukkan diferensiasi genetik yang lebih besar dibandingkan pasangan ras simpatrik *Mikania*–*Leucas*. Kedua pasangan ras menunjukkan isolasi habitat yang kuat, tetapi hanya pasangan ras *Dicliptera*–*Mikania* yang menunjukkan penghalang tambahan yang bekerja pada tahap lebih lanjut, termasuk penurunan keberhasilan kawin, penurunan daya tetas telur, dan penurunan kelangsungan hidup hibrida F_1_. Temuan kami menunjukkan bahwa penghalang reproduktif tambahan dapat berevolusi bahkan di tengah adanya isolasi habitat yang sangat kuat selama divergensi ras inang. Alih‐alih menggantikan pentingnya penghalang awal yang berasosiasi dengan inang, penghalang yang bekerja pada tahap lanjut dapat terakumulasi dan berkontribusi terhadap stabilitas isolasi reproduktif. Dengan demikian, kekuatan dan dinamika penghalang yang bekerja pada tahap lanjut dapat bergantung pada lintasan evolusioner masing‐masing pasangan ras inang, yang mencerminkan beragam jalur berlangsungnya spesiasi pada serangga yang berasosiasi dengan inang.

## 1 Introduction

The development of barriers to gene exchange among conspecific populations, leading to reproductive isolation (*RI*), is the key process underlying speciation (Dobzhansky 1951; Mayr 1959; Coyne and Orr 2004; Gavrilets 2004; Westram et al. 2022). Towards the completion of speciation, multiple barriers can evolve and become coupled, ultimately leading to complete isolation (Butlin and Smadja 2018; Kulmuni et al. 2020; but see Barton 2020; Servedio and Hermisson 2019; Barraclough 2024). This continued divergence ensures the diverging lineages remain separate, reducing the possibility of speciation reversal (Seehausen 2006; Grabenstein and Taylor 2018). In many systems, reproductive isolation accumulates unevenly, with some barriers appearing early or faster, whereas others emerge later or slower, and vary in their strength and importance (Nosil et al. 2017; Nosil 2021). Understanding speciation therefore requires identifying not only the barriers that first initiate divergence, but also examining whether additional barriers subsequently evolved, as well as the timing and tempo of their emergence. Although substantial progress has been made in identifying the first barriers to gene flow, the transition process from partial to strong reproductive isolation remains less well understood, especially the conditions under which different barriers accumulate and become coupled (Kulmuni et al. 2020).

Studies across taxa have shown that different *RI* components can exhibit different temporal dynamics. For example, in *Drosophila*, premating isolation evolves rapidly and generally faster than intrinsic postzygotic isolation, whereas postmating‐prezygotic (PMPZ) isolation appears to evolve at an intermediate rate, faster than hybrid inviability but slower than premating isolation (Turissini et al. 2017; Matute and Cooper 2021). Habitat and sexual isolation also appear to evolve early, whereas intrinsic postmating barriers may be more important for completing speciation in *Littorina* snails (Stankowski et al. 2020; Johannesson et al. 2024), birds (Uy et al. 2018; Ålund et al. 2024), and sticklebacks (Stelkens et al. 2010; Lackey and Boughman 2017), although intrinsic postzygotic isolation may accumulate earlier than previously assumed in cichlid fish (Rometsch et al. 2020). In *Timema* stick insects, sexual isolation evolves earlier and accumulates more gradually, whereas habitat isolation becomes more pronounced across species pairs (Nosil et al. 2007, 2024). In plants, by contrast, prezygotic barriers often contribute strongly to total *RI*, although PMPZ and postzygotic barriers can also be important depending on the system (Briscoe Runquist et al. 2014; Ma et al. 2016; Karrenberg et al. 2019; Shang et al. 2020), with PMPZ barriers being stronger than any individual postzygotic barrier (Christie et al. 2022). Despite this variation, general predictions can be made based on the initial driver of reproductive isolation. Seehausen et al. (2014) proposed that when speciation is driven by divergent selection, prezygotic and extrinsic postzygotic barriers are expected to evolve first, followed by intrinsic postzygotic barriers. This is because divergent selection can directly generate ecological or mating differences that reduce gene flow, whereas intrinsic postzygotic barriers generally require further genetic divergence.

This prediction is particularly relevant to phytophagous insects, where divergent host use can directly generate several early forms of *RI*. Because host plants function simultaneously as food resources, mating sites, and oviposition sites, divergence in host preference can generate habitat isolation and assortative mating, whereas host‐associated performance trade‐offs can generate immigrant inviability and reduced fitness through mismatched host preference, host‐finding ability, or performance on parental host plants (Bush 1969; Diehl and Bush 1984; Berlocher and Feder 2002; Drès and Mallet 2002; Matsubayashi et al. 2010). Consistent with this expectation, many host‐associated insect systems show strong host fidelity, host‐associated mating, temporal isolation, immigrant inviability, or reduced hybrid performance (Craig et al. 1993, 1997, 2001; Feder et al. 1994; Funk 1998; Via 1999; Via et al. 2000; Hawthorne and Via 2001; Emelianov et al. 2001, 2003; Pappers et al. 2002; Linn Jr et al. 2003, 2004; Egan and Funk 2009; McBride and Singer 2010; Lackey et al. 2023).

However, despite extensive work on host‐associated divergence, the accumulation of *RI* after early host‐associated isolation remains poorly understood. Most studies have focused on the origin of host preference, habitat isolation, or immigrant inviability, whereas fewer studies have tested whether additional sexual, postmating‐prezygotic, or postzygotic barriers accumulate after strong host‐associated isolation has already evolved (Egan and Funk 2009; Matsubayashi et al. 2010; Forbes et al. 2017). Existing examples suggest that host‐associated premating barriers and immigrant inviability often arise early, whereas later barriers vary more in their timing and mechanism. Ecologically mediated postzygotic isolation can appear even among host races or divergent populations (Via 1999; Craig et al. 1997; Via et al. 2000; Linn Jr et al. 2004; Egan and Funk 2009). By contrast, intrinsic postmating‐prezygotic or postzygotic barriers are less predictable. They may arise among more divergent host races, geographically structured populations, or closely related species, but are often weak or absent in early host‐race comparisons (Sheldon and Jones 2001; Matsubayashi et al. 2010; Rull et al. 2012; Tadeo et al. 2015; Fazalova et al. 2018). This raises a further question about the role of initial barrier strength. Strong barriers may occasionally appear early in divergence, although more often *RI* evolves slowly (Soltis et al. 2003; de Vos et al. 2020). If habitat isolation is incomplete, residual inter‐racial contact may allow selection against migrants or hybrids, reinforcement, or postmating barriers to evolve. If habitat isolation is nearly complete, however, gene flow may be reduced so strongly that later barriers are weakly selected or rarely expressed. Thus, it remains unclear whether strong early habitat isolation limits the opportunity for later barriers to evolve, or whether additional sexual and postmating barriers can still accumulate as host races continue to diverge.

In phytophagous ladybird beetles, studies of the *Henosepilachna vigintioctomaculata* species complex (Coleoptera: Coccinellidae: Epilachnini) have revealed that both sexual and postmating barriers can contribute to reproductive isolation, but most evidence comes from comparisons among closely related species (Katakura et al. 1981, 1989; Katakura and Hosogai 1994; Katakura 1997; Matsubayashi and Katakura 2007, 2009; Kuwajima et al. 2010; Matsubayashi et al. 2017). In these species‐level comparisons, barriers such as sexual isolation, reduced egg hatchability, conspecific sperm precedence, gametic isolation, and hybrid fitness reduction can occur, although their relative importance varies among species pairs. By contrast, studies of host‐associated divergence within this group suggest that early isolation may be dominated by host fidelity (Hirai et al. 2006).

*Henosepilachna diekei* Jadwiszczak & Węgrzynowicz provides a separate host‐race system for examining whether later‐acting barriers can accumulate within host‐associated divergence. Across its distribution in Southeast Asia, *H. diekei* consists of six host‐specialised races known to date, which utilise plants belonging to three families (Acanthaceae, Asteraceae, and Lamiaceae) and four genera (*Dicliptera, Mikania, Plectranthus*, and *Leucas*) (Fujiyama et al. 2013; Matsubayashi et al. 2015, 2016). In a sympatric host‐race pair in West Java, Indonesia, comprising one race feeding on Asteraceae: *Mikania micrantha* Kunth (referred to as the *Mikania* race) and the other on Lamiaceae: *Leucas lavandulifolia* Sm. (referred to as the *Leucas* race), extremely divergent host plant preferences, performances, and host‐associated assortative mating were the only major barriers detected (Matsubayashi et al. 2011, 2019; Matsubayashi, Kahono, Hartini, et al. 2013; Matsubayashi, Kahono, and Katakura 2013). These findings suggest that habitat isolation and immigrant inviability, manifested as host plant specialisation, act as the first barrier and critical driving force in initiating speciation in phytophagous ladybird beetles (Matsubayashi et al. 2011) before the evolution of sexual or postmating barriers. However, it remains unclear whether later‐acting barriers can already accumulate within host‐race divergence during the early stages of speciation, particularly when very strong early‐acting barriers such as habitat isolation have already evolved.

In Mt. Patuha, West Java, populations feeding on the native plant, Acanthaceae: *Dicliptera canescens* Nees (referred to as *Dicliptera* race), have been recognised as a distinct host race because they are geographically isolated from other host races at high elevation (~1600 masl), occur as small populations and display a unique combination of body shape and elytral maculation not found in other known host races (Matsubayashi et al. 2015, 2016). Because phenotypic divergence can sometimes covary with genetic divergence during population divergence, especially for traits associated with reproductive isolation or lineage differentiation (Schluter and Rieseberg 2022; Freeman et al. 2023), these ecological, geographical, and morphological features suggest that the *Dicliptera* race may represent a more differentiated host‐race lineage, although this inference requires genetic confirmation. By contrast, the *Mikania* race feeds on *Mikania micrantha*, a recently introduced host plant in Java, and has been suggested to represent a relatively recent host‐race lineage. Therefore, comparing the *Dicliptera–Mikania* race pair with the previously studied *Mikania–Leucas* race pair allows us to examine *RI* across two putative levels of host‐race divergence within *H. diekei*: a less differentiated pair characterised mainly by strong habitat isolation, and a potentially more differentiated pair that may reveal whether additional sexual or postmating barriers accumulate after early habitat isolation.

In this study, we investigated the presence of reproductive barriers beyond the first barrier (habitat isolation) in *H. diekei* and discuss their role in the population divergence process in phytophagous ladybird beetles. We first examined the microspatial distribution of host plants and beetles and assessed the genetic relationship between the *Dicliptera* race and previously known host races, including whether the *Dicliptera–Mikania* race pair is more genetically differentiated than the previously studied *Mikania–Leucas* race pair. To address whether additional barriers have accumulated in this pair, we conducted a series of laboratory and field‐cage experiments to examine and estimate various pre‐ (habitat isolation and sexual isolation) and postmating barriers (postmating‐prehatching or reduced egg hatchability and postmating‐posthatching or F_1_ hybrid inviability) between the *Mikania* race and the *Dicliptera* race. By comparing these barriers with those previously reported for the *Mikania–Leucas* race pair, we specifically ask whether strong early host‐associated habitat isolation is accompanied by the accumulation of additional sexual and postmating barriers as host‐race divergence proceeds. We then discuss how these barriers may evolve, the dynamics of their accumulation, and their general importance in the speciation process of phytophagous ladybird beetles.

## 2 Materials and Methods

### 2.1 Fieldwork

#### 2.1.1 Microspatial Distribution of Beetles and Host Plants

The distributions of the two host plant species, *D. canescens* and *M. micrantha* , as well as the ladybird beetles, *H. diekei*, that feed on them, were surveyed in an area of approximately 10 × 10 km^2^ in the vicinity of Bandung and Ciwidey, West Java, Indonesia, from September 2019 to February 2020. Beetles occurring within a continuous patch of a single host plant species were considered a single population. Coordinates (WGS84) of localities were taken using a GPS device or determined as accurately as possible from a map. The map was reconstructed from a digital elevation model of the island using QGIS 3.22.6 based on the Global30‐Arc‐Second Elevation Data (GTOPO30) from the U.S. Geological Survey and Seamless Digital Elevation Model 0.27‐arcsecond (DEMNAS) from the Indonesian Geospatial Information Agency.

#### 2.1.2 Beetle Collection and Rearing

**FIGURE 1:**
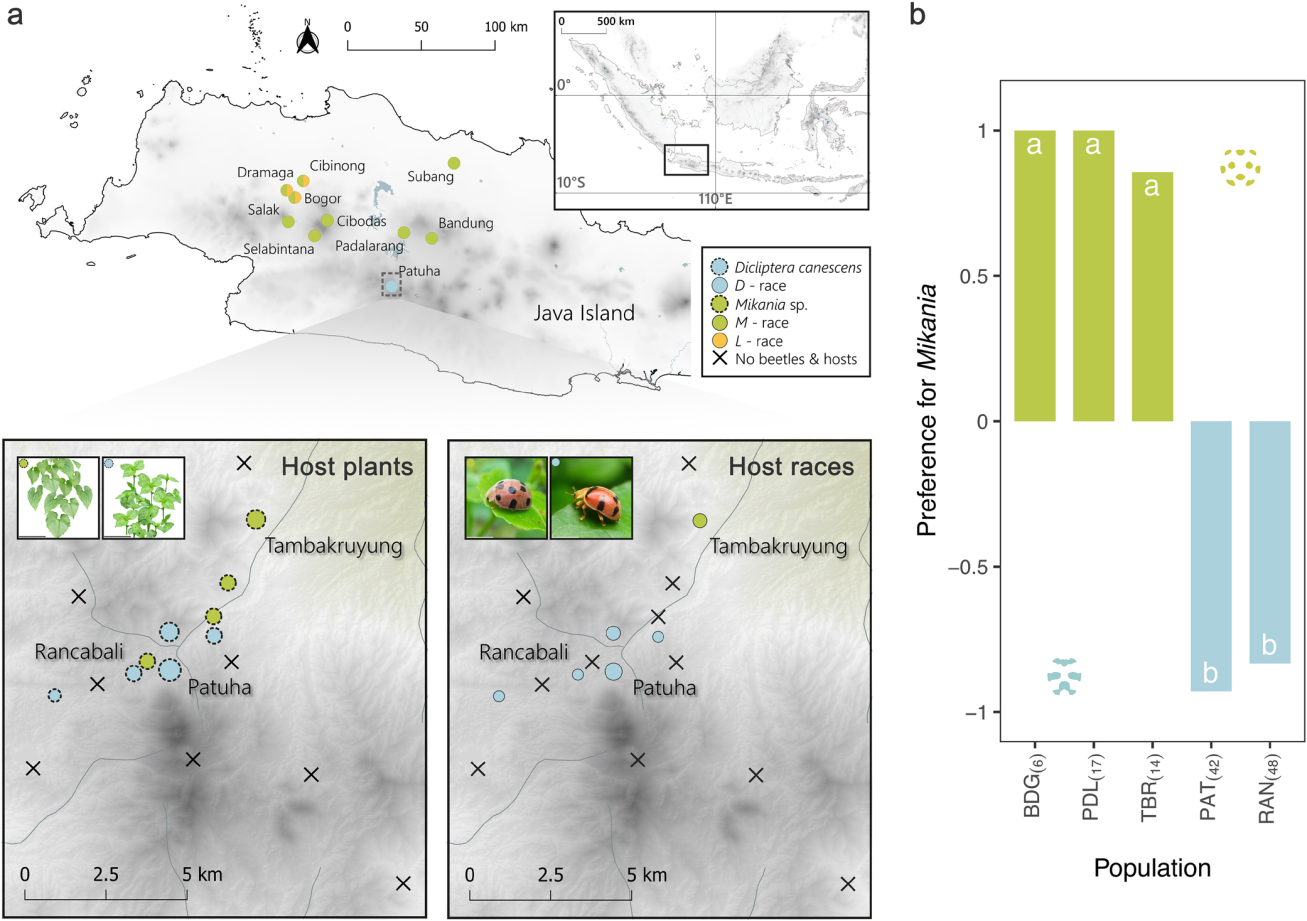
Spatial distribution of *Henosepilachna diekei* host‐race populations in West Java, Indonesia (a) and results of the host preference test for the two host plants, *Mikania micrantha* and *Dicliptera canescens* (b). Dotted grey outline denotes the 10 × 10 km^2^ areas surveyed in southern Bandung. Host preference responses in dual‐choice experiments using field‐collected beetles from five localities did not differ by sex. Preferences are shown as proportion of beetle individuals displaying each feeding response, with signs indicating the direction of preference. The number of individuals tested (*n*) is shown for each bin. Different letters on each bin indicate significant differences (*p <* 0.05). BDG, Bandung; PAT, Patuha; PDL, Padalarang; RAN, Rancabali; TBR, Tambakruyung.

Beetles were collected during the field survey mentioned above. Beetles collected in the wild on *D. canescens* were defined as *Dicliptera* race (*D*‐race), and those collected on *M. micrantha* as *Mikania* race (*M*‐race). Adult beetles of *H. diekei* were collected from two *D*‐race populations (Patuha and Rancabali) and three *M*‐race populations (Padalarang, Bandung and Tambakruyung) (Figure 1). After collection, beetles were transferred to the laboratory, separated by host race and population, and reared in groups of 5–10 individuals in plastic boxes (7 cm × 7 cm × 2.5 cm) with moistened tissue paper lining the bottom. Within each box, males and females were allowed to mate freely.

Field‐collected beetles from all populations were used in the host preference test. To establish laboratory stock populations for subsequent experiments, wild‐caught *M*‐race beetles from Padalarang and *D*‐race beetles from Patuha were maintained separately by race and used to produce within‐race offspring stocks. Within each stock population, beetles were maintained under the same conditions and allowed to mate freely, and egg batches were collected daily.

Eggs from these within‐race stock populations were separated, and newly emerged larvae were raised individually to adulthood under controlled room conditions (20°C) and approximately 12 h light: 12 h dark. These laboratory‐reared adults (first‐generation, F_1_) were used in all subsequent experiments. Fresh leaves of the original host plants were served daily. To supply foliage throughout the experiments, *D. canescens* was collected weekly from its original location in Patuha, while *M. micrantha* was collected from the vicinity of the IPB University campus (Dramaga, West Java). We note that a full common garden generation was not implemented in this study, and thus some plastic or transgenerational environmental effects may not have been completely removed. However, such an approach is technically constrained in this system because both host races exhibit absolute host specificity and cannot be successfully reared on alternative host plants (Matsubayashi et al. 2011, 2013; Matsubayashi et al. 2019).

### 2.2 Molecular Analysis

#### 2.2.1 Genetic Relationships Among Host Races in Java Island

Phylogenetic relationships among host races were examined using partial sequences of the mitochondrial DNA NADH‐dehydrogenase subunit 2 (ND2) gene of *D*‐race beetles in West Java. Eleven adult *D*‐race beetles were collected and ND2 sequences were analysed following the method described in Fujiyama et al. (2013). To elucidate the phylogenetic position of the *D*‐race, we compared its haplotypes with previously reported haplotypes of two other host races occurring on *M. micrantha* and *L. lavandulifolia* from nine populations across three regions (West Java, Central Java, and East Java; GenBank AB830888—AB830902) (Fujiyama et al. 2013). Collected sequences were aligned with Clustal W in MEGA X (Kumar et al. 2018) and adjusted visually. Median‐joining haplotype network was constructed using Network ver. 10.2.0.0 software (Bandelt et al. 1999). Net genetic distance (*D*_xy_) and fixation index (*F*_ST_) between three host races from three geographic areas were calculated using MEGA X and Arlequin ver. 3.5.2.2 (Excoffier and Lischer 2010) based on ND2 sequences only.

### 2.3 Measurements of Reproductive Barriers in Laboratory Experiments

All laboratory experiments were performed between September 2019 and March 2020 in the Animal Experimental House, Department of Biology, FMIPA, IPB University.

#### 2.3.1 Host Preference Test (Habitat Isolation)

Feeding preferences of wild‐caught adult beetles were evaluated for *M. micrantha* and *D. canescens* using a choice experiment in the laboratory. A total of 127 wild‐caught individuals were tested, comprising three *M*‐race populations (Bandung, *n* = 6; Padalarang, *n* = 17; Tambakruyung, *n* = 14) and two *D*‐race populations (PAT, *n* = 42; RAN, *n* = 48). Beetles were tested on the following day after their collection. Each beetle was placed individually into a plastic box (9 cm × 5 cm × 1.5 cm) containing a piece of fresh leaf with an area approximately 12 cm^2^ from each of *D. canescens* and *M. micrantha* . Beetles were allowed to feed freely for 24 h. Feeding responses were recorded based on whether the individual ate only on *Dicliptera*, only on *Mikania*, on both host plants, or on neither. After the test, all leaf pieces were taped to white paper and scanned using EPSON Scanner V39 (Epson, Tokyo, Japan). Leaf area consumed was measured from the digital images using ImageJ 1.52 software (Schneider et al. 2012).

Significance of differential host preference between host races was assessed using GLMs with quasi‐binomial error and logit link, including “race”, “sex” of the individual and the interaction (“race × sex”) as explanatory variables and the number of individuals preferring each host (“preference”) as the response variable. We also tested differences of the preference between populations within each host race using “population”, “sex” of the individual, and the interaction (“population × sex”) as explanatory variables.

#### 2.3.2 Non‐Choice Mating Test (Sexual Isolation)

We used virgin adults from the stock population to check sexual isolation in a non‐choice mating test. All assays were conducted using first‐generation (F_1_) laboratory‐reared adults derived from wild‐caught parents (*M*‐race: Padalarang; *D*‐race: Patuha; see Beetle Collection and Rearing section). One male and one female from two types of adults (*D*‐race and *M*‐race) were placed together in a small box (5.5 cm × 5.5 cm × 2.5 cm). In this experiment, four mating combinations were crossed (maternal type first: *D* × *D*; *D* × *M*; *M* × *D*; and *M* × *M*). Mating behaviour (male attempt and successful mating) was observed directly for 60 min. Mating was regarded as successful when it lasted for more than 30 min after genital contact.

To assess the factors determining the number of mating attempts and successes, we used GLMs with binomial error and logit link, including “female” type, “male” type, and the interaction (“female × male”) as explanatory variables and the number of attempts/successes or failures as the response variable.

#### 2.3.3 Egg Hatchability Test (Postmating‐Prehatching Isolation)

Crosses between virgin females and males from both races were conducted using first‐generation (F_1_) laboratory‐reared adults derived from wild‐caught parents (see Beetle Collection and Rearing section), originating from single populations per host race (*M*‐race: Padalarang; *D*‐race: Patuha). Virgin females and males of both races were allowed to mate once, after which females were kept individually in plastic cups (Ø 5.5 cm, V = 35 mL) for oviposition in the female natal host plant. In this experiment, four mating combinations were tested, including intra‐racial matings (*D* × *D* and *M* × *M*) and inter‐racial matings (*D* × *M* and *M* × *D*). Newly laid egg batches were collected daily, and the number of eggs, hatched larvae, and hatching duration were recorded for each female.

The number of eggs per batch, the hatching rate, and the hatching duration among intra‐ and inter‐racial mating were analysed using GLMMs. A quasi‐binomial error structure with a logit link was used for egg hatchability and a quasi‐Poisson error structure with log link function for the number of eggs produced and hatching duration. In these models, “female” type, “male” type, and the interaction (“female × male”) were included as explanatory variables, and the number of eggs produced, the number of eggs hatched or failed to hatch, and the period until hatching were used as the response variables. We included the “family” nested within “female” type as a random effect. We used the term “family” to indicate progeny produced by females after copulation with a single male (full‐sib brood).

#### 2.3.4 Larval Performance Test (Postmating‐Posthatching Isolation)

Growth performance of larvae from both host races and their reciprocal F_1_ hybrids was tested on *D. canescens* and *M. micrantha* to measure relative fitness on the two host plants. Hatched larvae from egg hatchability tests were used in this test. From one to several egg masses laid by each female beetle, at most 20 newly hatched larvae from each mother were obtained and divided randomly into two groups of equal size. When an odd number of larvae hatched from a single egg mass, one larva was arbitrarily assigned to either group. One group of larvae was reared on leaves of *D. canescens* and the other on *M. micrantha* (split‐brood design) in plastic cups (Ø 5.5 cm, V = 35 mL). Around 10 to 13 mothers were used per crossing combination (10, 13, 11, for *M* × *M, M* × *D*, and *D* × *D*, respectively), resulting in 200, 160, and 200 larvae for respective groups. An exception for the *D* × *M* combination, in which only 3 females were used, yielded fewer than 10 larvae each (a total of 20 larvae), due to the relatively low hatching rates. Newly hatched larvae were placed on leaves within a day of emergence and reared individually. Fresh leaves were supplied daily, and the development of the larvae (1st instar survival, 2nd instar survival, and survival to adulthood) and their initial acceptance of the respective host plants were monitored daily.

The association between fitness components (larval acceptance, survival to the second instar, and ability to reach adulthood) and both host plant and crossing type was analysed using GLMMs with a quasi‐binomial error structure and logit link function. We specifically tested (i) differences in fitness between host plants within each crossing type (i.e., performance on natal versus alternative host plants), and (ii) differences among crossing types within each host plant. In these models, “female” type, “male” type, “food”, and the interaction (“female × male × food”) were included as explanatory variables and the number of larvae successfully reaching a given developmental stage (1st instar, 2nd instar, pupation/adulthood) or failing to do so was used as the response variable. The “family” effect was included as random effect nested within the “female” type.

### 2.4 Assortative Mating and Reproductive Isolation in Field Cage Experiments

#### 2.4.1 Mate Choice in the Absence and Presence of Host Plants

Field‐cage experiments were conducted in the greenhouse facility, Department of Biology, Faculty of Mathematics and Natural Sciences (FMIPA), IPB University. Mate choice was conducted using a two‐way multiple‐choice design, with females and males from the two host races in field cages set up on bare soil (4 × 4 × 2 m^3^), where all grasses had been removed. The cages were covered with nylon netting. Ten potted *D. canescens* and 10 potted *M. micrantha* plants were arranged either in a separate design (S‐cage) or a checkerboard design (C‐cage), simulating parapatric and sympatric host patch distributions, respectively (see Matsubayashi, Kahono, and Katakura 2013). Fifteen newly emerged virgin females and 15 males of each race were marked and released into each respective cage (60 individuals in total), at a ground‐level release point at the centre of the cage. Females were released a day prior to males and allowed to disperse freely to their preferred plant. Individuals were marked using unique combinations of dot positions and ink colours applied with permanent markers. Observations were conducted twice daily for 1 h per session, over six consecutive days, to standardise observation effort. During observations, we recorded the location of host plant on which each beetle was sighted and the identity of mating pairs. The two spatial treatments (separate and checkerboard designs) were conducted simultaneously in two cages under identical greenhouse conditions. Each treatment was performed once; thus, cage‐level replication was not included. However, behavioural observations were replicated through repeated observations of individually marked beetles over time. The number of sightings on each host plant was analysed using a GLM with binomial error structure and logit link function.

We also conducted mate choice tests in the laboratory to test the effect of the absence of host plants. Mate choice tests were conducted using a one‐way male choice design. Six virgin females for each race were released into a large plastic box (9 cm × 7.5 cm × 3.5 cm). After dispersion of the females (30 min), six virgin males of both races were released. Observations were terminated once three of six potential pairs had been established (Casares et al. 1998). Mating was regarded as successful when a female accepted the genitalia of a male. This design allows males to choose among multiple potential mates in the presence of competitors, providing a more natural behavioural context than the non‐choice assay. Together with the laboratory mating assays (non‐choice and mate‐choice tests), the field‐cage experiment enables comparison of sexual isolation across controlled and semi‐natural conditions, allowing us to evaluate context‐dependent reproductive isolation.

The intensity of assortative mating, measured as the number of intra‐ versus inter‐racial mating pairs, was compared between field‐cage observations and mate choice tests conducted in the absence of host plants. This was analysed using GLMs with binomial error structure and logit link function. We tested the effect of “male” type, the presence/absence of “host”, and the interaction (“male × host”) as explanatory variables and the number of intra−/inter‐racial mating instances as the response variable. This model tests whether males of different host races differ in their tendency to form intra‐ versus inter‐racial mating pairs under different environmental conditions. We also analysed whether different host arrangements influenced mating inferences. Here, we included “male” type and host “arrangement” as explanatory variables.

#### 2.4.2 Host Fidelity and Dispersal

The dispersal distance was calculated based on individual sighting records by adding the distances between the plant pots on which an individual was observed during the field‐cage experiments (S‐cage and C‐cage). Following the assumptions of Matsubayashi, Kahono, and Katakura (2013), dispersal distance was treated as a per‐day (24 h) metric, ignoring observations of beetles on the cage netting. The frequencies of intra‐ and inter‐host migrations were also calculated.

To detect host fidelity, we assessed which factors explained beetle sightings, migration, and dispersal distance in the field cage by using “race”, “sex” of individual, “arrangement” of host plants, and the interactions (“race × sex”, “race” × “arrangement”, “sex” × “arrangement”, “race” × “sex” × “arrangement”) as explanatory variables. The number of sightings on each host plant, the number of inter−/intra migration, and dispersal distance were used as response variables, respectively. These responses were analysed using GLMs with binomial error structure and logit link function for migration events, and quasi‐Poisson error structure with log link function for dispersal distance.

### 2.5 Statistical Analyses

We applied generalised linear models (GLMs) and generalised linear mixed models (GLMMs) using R‐4.1.2 (R Development Core Team 2021) to test the differences in each measure of reproductive isolation.

We first estimated the effect of each explanatory variable and then tested the significance of the effect of each factor using Wald *χ* ^2^ test in the package “car” (Bolker et al. 2008; Fox and Weisberg 2019). In the case of pairwise comparisons, the significance of each comparison was determined using the “multcomp” package with Holm‐adjusted method. In the model fitting for each response variable, appropriate error distributions and link functions were applied as described in the relevant sections above. Quasi‐binomial and quasi‐Poisson error structures were used due to overdispersion in the data.

### 2.6 Quantification of Reproductive Isolation

The strength of reproductive isolation between *M*‐race and *D*‐race was evaluated by calculating the strength of each individual barrier at each stage and the total strength of *RI*. *RI* was calculated separately for each host race as directional estimates. For mating and postmating barriers, *RI* was calculated for each reciprocal cross direction (maternal race first), representing the degree to which females of each host race are isolated from the alternative race. For habitat isolation, *RI* represents race‐specific estimates of encounter probability derived from host preference. The absolute strength of reproductive isolation was calculated with the method in Sobel and Chen (2014) as follows:

$$\mathrm{RI}=1-2\times \left(\;\frac{H}{H+C}\right)$$

where *RI* is the absolute strength of the isolating barrier, and *H* and *C* are the frequencies of heterospecific and conspecific events, respectively. This *RI* varies from −1 to 1, where positive values indicate reduced gene flow between races (i.e., reproductive isolation), values near 0 indicate no isolation, and negative values indicate excess heterospecific interactions. Confidence intervals for *RI* estimates were obtained by bootstrap resampling (10,000 iterations) of the underlying data. To facilitate comparison across studies, all *RI*estimates were additionally coded using the standardised RIO reporting framework (Walker et al. 2026).

To assess the relative contribution of each barrier to the total isolation experienced between host races, we calculated it using the following formula (Coyne and Orr 1989; Ramsey et al. 2003):

$${\mathrm{RI}}_{\text{total}}=\mathrm{pre}+\left(1-\mathrm{pre}\right)\times \text{post}$$

where ${\mathrm{RI}}_{\text{total}}$ is the total strength of reproductive isolation as a linear, sequential sum of premating (pre) and postmating (post) isolation, which discounts later‐acting barriers by those that have already acted.

The mathematical expression above holds true in the normal situation. For the earliest‐acting barrier (i.e., habitat isolation), we assumed both habitats (in our case, host plants) were of equal quality and abundance. To explore how environmental disturbance could affect the strength of reproductive barriers and their cumulative contribution to total *RI*, we recalculated *RI* under scenarios where the absolute contribution of habitat isolation (${\mathrm{RI}}_{\mathrm{HI}}$) was progressively reduced. This reflects the expectation that environmental disturbance may weaken habitat‐based isolation. Because later‐acting barriers only affect individuals that pass through earlier barriers, their absolute contributions depend on the strength of preceding barriers. Thus, changes in habitat isolation may alter the relative importance of subsequent barriers.

We simulated moderate, strong, and extreme disturbance by reducing the absolute contribution of habitat isolation to half, a quarter, and zero of its value under normal conditions, respectively:

$${\mathrm{RI}}_{\mathrm{HI}\;\text{moderate}}={\mathrm{RI}}_{\mathrm{HI}}\times 0.5$$

$${\mathrm{RI}}_{\mathrm{HI}\;\text{strong}}={\mathrm{RI}}_{\mathrm{HI}}\times 0.25$$

$${\mathrm{RI}}_{\mathrm{HI}\;\text{extreme}}={\mathrm{RI}}_{\mathrm{HI}}\times 0$$

To facilitate comparison across host‐race pairs at different levels of differentiation, we also calculated *RI* estimates for the *L*‐race and *M*‐race pair from West Java using published data from Matsubayashi et al. (2011) and Matsubayashi, Kahono, and Katakura (2013), which employed a largely comparable experimental design.

## 3 Results

### 3.1 Microspatial Distribution of Beetles and Host Plants

In our field survey, *Mikania micrantha* was widely distributed in relatively moist, semi‐open habitats with moderate sunlight, often spreading over other plants near streams. In contrast, *Dicliptera canescens* was observed in moist, shady and cooler habitats at higher altitudes, such as the foot of mountains, coffee plantations and roadside edges. No infestation of *M. micrantha* by *H. diekei* was observed at higher altitudes, and the nearest *M*‐race was located approximately 5 km (Mt. Tambakruyung) from the nearest *D*‐race population (Mt. Patuha) (Figure 1). Details of observation sites are presented in Table S1. Although the distributions of the two host plants partially overlapped, both *M*‐race and *D*‐race beetles were spatially separated and thus considered allopatric (Figure 1a).

### 3.2 Phylogenetic Position of *D*‐Race

**FIGURE 2:**
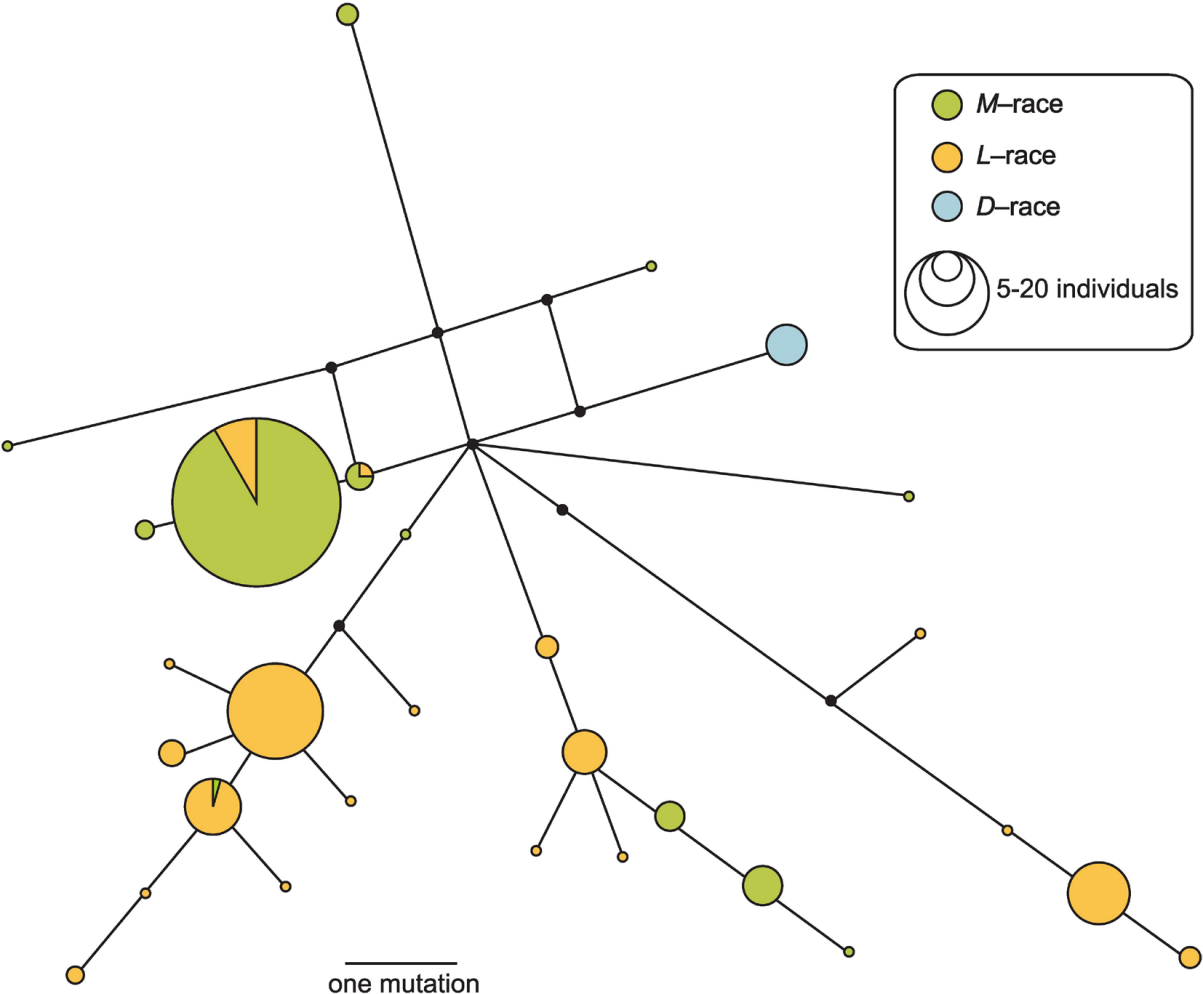
Haplotype networks based on mitochondrial *ND2* sequences of host races of *Henosepilachna diekei* in West Java. Haplotypes of the *M‐*race (occurring on *Mikania micrantha* ), the *L‐*race (on *Leucas lavandulifolia*), and the *D‐*race (on *Dicliptera canescens*) are denoted in green, orange, and blue, respectively. Circle sizes correspond to the number of samples. Missing haplotypes are represented as black dots.

All 11 individuals of the *D*‐race shared one unique haplotype (DDBJ accession number LC940254), distinct from the 15 unique haplotypes previously detected in other host races on Java based on ND2 sequences (Fujiyama et al. 2013). Population pairwise *F*_ST_ and *D*_XY_ values, estimated from ND2 sequences, indicated strong genetic structuring among host races, consistent with restricted gene flow (Table 1). In particular, differentiation between *D*‐race and *M*‐race in West Java was higher (*F*_ST_ = 1) than that between *L*‐race and *M*‐race in West Java (*F*_ST_ = 0.3627), suggesting stronger genetic isolation between *D*‐ and *M*‐races. Absolute genetic divergence (*D*_XY_) was relatively low overall, although still higher between *D*‐race and *M*‐race (*D*_XY_ = 0.0079) than between *L*‐race and *M*‐race (0.0049) in West Java. The haplotype network further demonstrated that *D*‐race haplotypes are clearly separated from those of the other host races (Figure 2).

**TABLE 1:** Average number of nucleotide substitutions (*D*_xy_: Upper‐right) and pairwise genetic fixation index (*F*_ST_: Lower‐left) estimated from ND2 sequences, among host races of *Henosepilachna diekei* from four localities on Java Island, Indonesia.

*F*_ST_/*D*_xy_	Central (*L*)	East (*M*)	West (*L*)	West (*M*)	West (*D*)
Central (*L*)	—	0.0159	0.0157	0.0145	0.0130
East (*M*)	**0.8113**	—	0.0095	0.0101	0.0116
West (*L*)	**0.8292**	**0.3545**	—	0.0049	0.0081
West (*M*)	**0.9810**	**0.6631**	**0.3627**	—	0.0079
West (*D*)	**0.9760**	**0.6738**	**0.5626**	**1.0000**	—

*Note:* Significant values of *F*_ST_ between populations (*α* ≤ 0.05) are represented in bold. *M* = on *Mikania micrantha* , *L* = on *Leucas lavandulifolia*, *D* = on *Dicliptera canescens*.

The variation in ND2‐based genetic differentiation among host races across geographic regions suggests possible differences in the degree of mitochondrial divergence among race pairs. In this context, the *L*–*M* and *D*–*M* race pairs represent two distinct levels of ND2‐based differentiation, providing a useful framework for examining how additional reproductive barriers may evolve following the establishment of strong habitat isolation. Because this inference is based on a single mitochondrial marker, it should be interpreted cautiously and may not necessarily reflect genome‐wide patterns of differentiation.

### 3.3 Host Preference (Habitat Isolation)

The laboratory feeding assay revealed that both host races exhibited very clear preferences for their natal host plant (Figure 1b). *D*‐race individuals chose only *D. canescens* (92% of 42 individuals from Patuha; 83% of 48 individuals from Rancabali) (Table S2). Conversely, the *M*‐race chose only *M. micrantha* (100% of 17 individuals from the Padalarang population; 100% of 6 individuals from the Bandung population; 86% of 14 individuals from the Tambakruyung population). A small proportion of both host races demonstrated null‐choice (8% of the *D*‐race from Patuha; 17% of the *D*‐race from Rancabali; 14% of the *M*‐race from Tambakruyung). The preference was explained by a strong effect of “race” regardless of the collection site in GLM (estimate = 54.85 ± 0.67, *p* < 0.0001; Table S3). No significant effects of “population” and “sex” were detected in same‐race comparisons (Table S3).

### 3.4 Non‐Choice Mating (Sexual Isolation)

**FIGURE 3:**
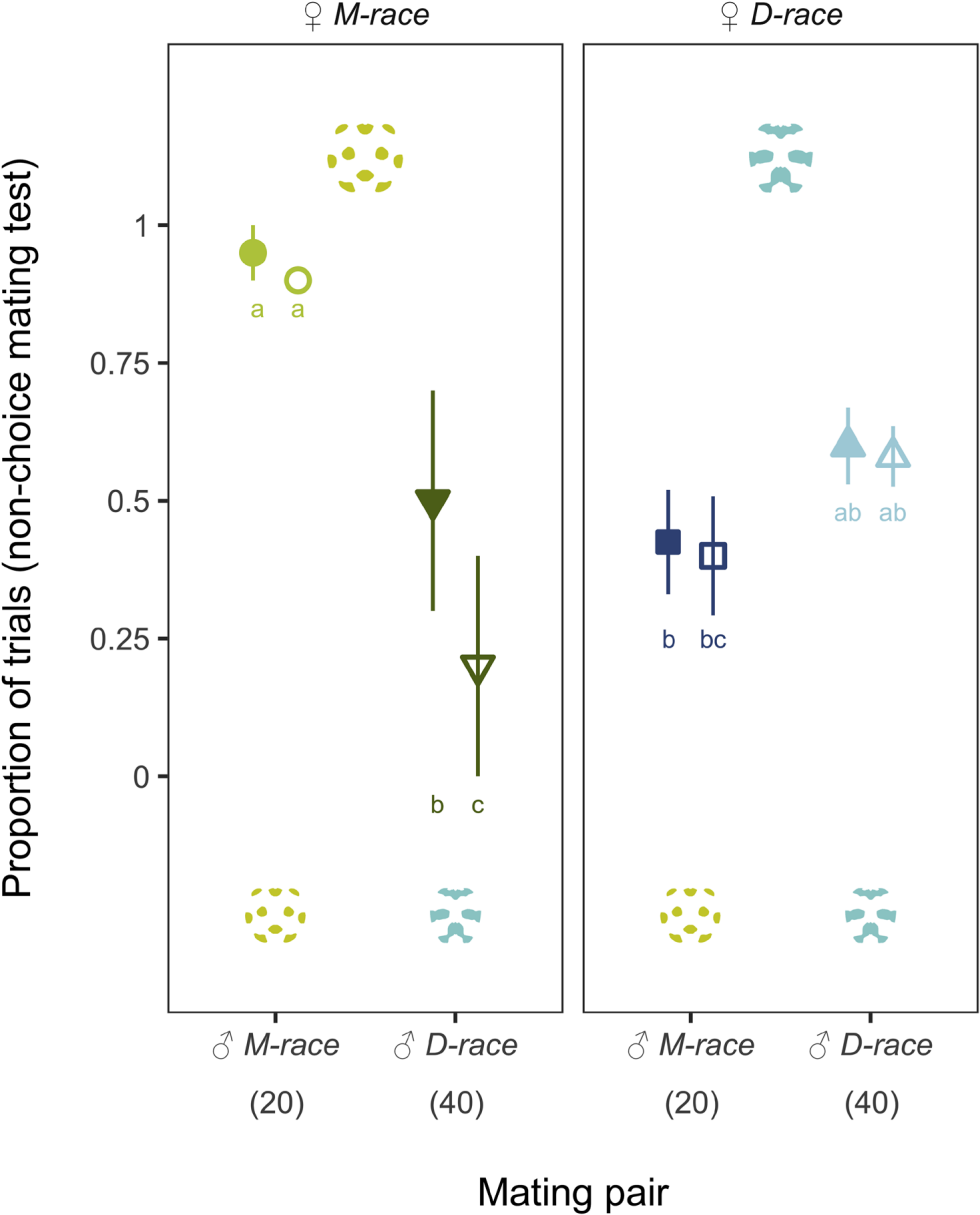
The degree of sexual isolation between two *Henosepilachna diekei* host races, *M* (*Mikania*) and *D* (*Dicliptera*) ‐races inferred from non‐choice mating tests. The proportion of trials in which males initiated mating attempts towards females of each race is shown using closed symbols, and the proportion of trials resulting in successful copulation (lasting at least 30 min) is shown using open symbols (mean ± SEM). The number of replicates is shown on the axis. Different letters on each bin indicate significant differences among mating pairs (*p <* 0.05). Light blue triangles, *D × D*; dark blue squares, *D × M*; dark green inverted triangles, *M × D*; light green circles, *M × M*. In each combination, the first letter denotes the female race and the second letter denotes the male race.

The outcomes of the non‐choice mating test (maternal type first: 24 pairs of *D*‐race × *D*‐race, 16 pairs of *D*‐race × *M*‐race, 4 pairs of *M*‐race × *D*‐race, and 18 pairs of *M*‐race × *M*‐race), based on 40 replicate trials for *D* × *D* and *D* × *M*, and 20 replicate trials for *M* × *D* and *M* × *M*, showed a significant interaction effect between “female” and “male” types affecting both mating attempts and success (GLM: mating attempts, estimate = 3.76 ± 1.21, *p* = 0.0019; mating success, estimate = 4.39 ± 1.04, *p* < 0.0001; Tables S4, S5). Mating behaviour differed among cross types (Figure 3). In the *M*‐female direction, *M* × *M* pairs exhibited high mating activity (attempts: 0.95 ± 0.05; success: 0.90 ± 0.00), whereas inter‐racial *M* × *D* pairs showed substantially reduced mating attempts (0.50 ± 0.20) and especially low mating success (0.20 ± 0.20). In contrast, in the *D*‐female direction, mating attempts were more similar between cross types (*D* × *D*: 0.60 ± 0.07; *D* × *M*: 0.43 ± 0.09), although mating success was lower in inter‐racial crosses (*D* × *D*: 0.58 ± 0.06; *D* × *M*: 0.4 ± 0.11). Note that in general, fewer mating activities were observed in the *D*‐race compared to the *M*‐race, although this difference was not statistically significant (estimate = −2.43 ± 1.07, *p* = 0.0952; Table S4). Overall, *M*‐race males made fewer mating attempts and achieved lower mating success with *D*‐race females, whereas *D*‐race males showed no clear difference in mating attempts towards females of either race but reduced success in inter‐racial crosses (Figure 3).

### 3.5 Egg Hatchability (Postmating‐Prehatching Isolation)

**FIGURE 4:**
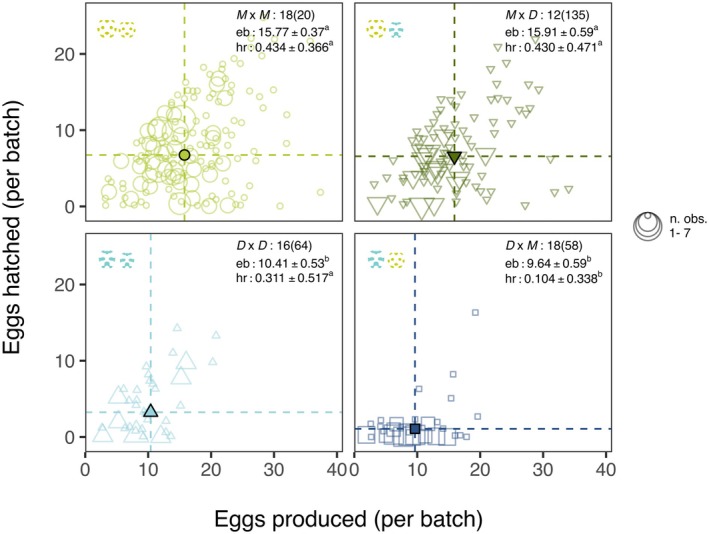
Hatchability of eggs produced from intra‐ and inter‐racial mating between host races of *Henosepilachna diekei*. The number of females used is shown with the total number of egg batches in parentheses. Each point represents a combination of eggs produced and eggs hatched per batch, and symbol size indicates the number of observations for each combination (n. obs.). Means are shown as closed symbols. Different superscript letters indicate significant differences among mating pairs (*p <* 0.05). Light blue triangles, *D × D*; dark blue squares, *D × M*; dark green inverted triangles, *M × D*; light green circles, *M × M*. In each combination, the first letter denotes the female race and the second letter denotes the male race. eb., number of eggs produced per batch (mean ± SEM); hr., hatching rate (mean ± SEM).

GLMM analysis showed that the “female” type explained most of the variation in the number of eggs produced per batch (estimate = 0.41 ± 0.09, *p* < 0.0001; Table S6), whereas no significant interaction between female and male types was detected. Mean egg production per batch was highest in *M* × *M* (15.77 ± 0.37) and *M* × *D* (15.91 ± 0.59) crosses, and lower in *D* × *D* (10.41 ± 0.53) and *D* × *M* (9.64 ± 0.59) crosses (Figure 4). Pairwise comparisons showed significant differences in the number of eggs produced between intra‐racial mate combinations (*D* × *D* vs. *M* × *M*, estimate = −0.42 ± 0.08, *p* < 0.0001) and between inter‐racial mate combinations with different maternal types (*D* × *D* vs. *M* × *D*, *D* × *M* vs. *M* × *D*, *D* × *M* vs. *M* × *M*; e.g., *D* × *M* vs. *M* × *D*, estimate = −0.49 ± 0.09, *p* < 0.0001; Table S6). No differences were detected between inter‐racial mate combinations that shared the same maternal type (*D* × *M* vs. *D* × *D*, estimate = 0.08 ± 0.10, *p* = 0.857; *M* × *D* vs. *M* × *M*, estimate = −0.02 ± 0.08, *p* = 0.857; Table S6; Figure 4).

Despite the smaller number of eggs produced in *D* × *D* compared to those from *M*‐race females (*M* × *M*, *M* × *D*), there were no significant differences in the hatching rate (e.g., *D* × *D* vs. *M* × *M*, estimate = −0.47 ± 0.23, *p* = 0.118; Table S7). Mean hatching rates were similar in *M*‐female crosses (*M* × *M*: 0.434 ± 0.366; *M* × *D*: 0.430 ± 0.471), whereas lower values were observed in *D*‐female crosses (*D* × *D*: 0.311 ± 0.517; *D* × *M*: 0.104 ± 0.338; Figure 4). In *D*‐race females, a slightly lower hatching rate was observed following the inter‐racial matings (*D* × *M* vs. *D* × *D*, estimate = 1.33 ± 0.39, *p* = 0.003; Figure 4). Complete GLM results for all pairwise comparisons are provided in Table S7. There were no differences in hatching duration, except that eggs from *D* × *M* had a significantly longer hatching period (Table S8).

### 3.6 Larval Performance (Postmating‐Posthatching Isolation)

**FIGURE 5:**
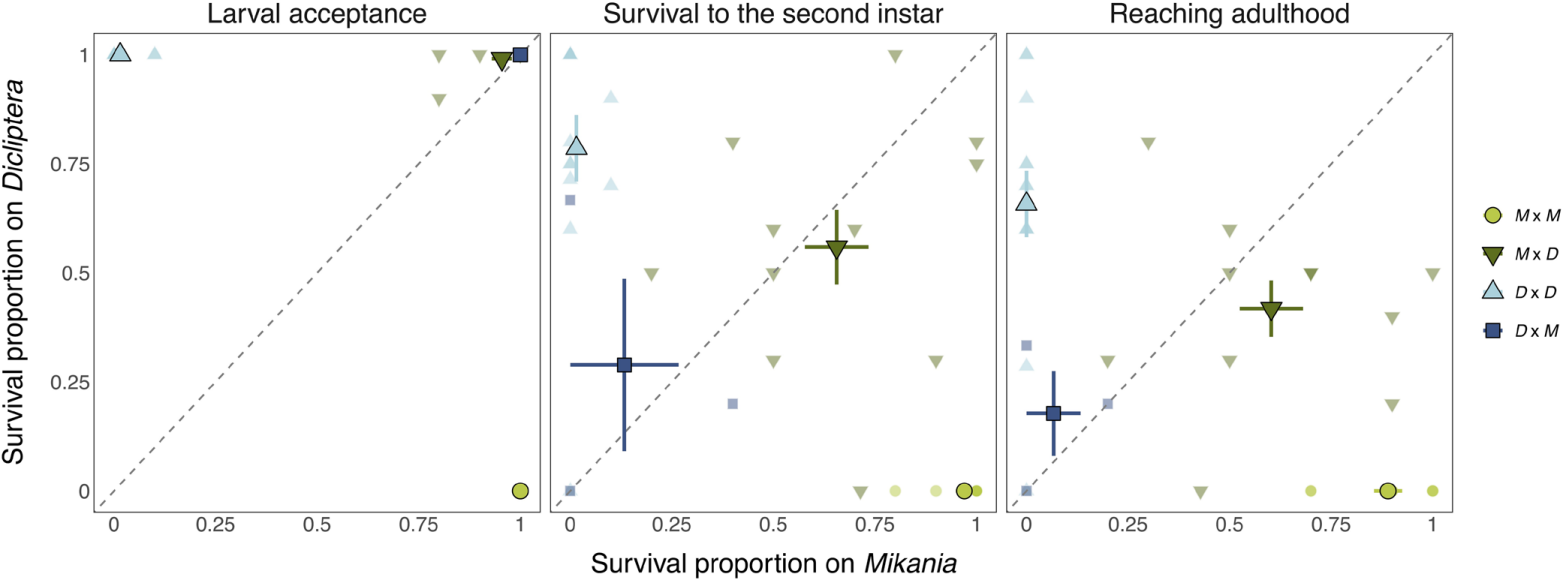
Host *×* family interaction plots of developmental traits at the family level for four *Henosepilachna diekei* larval progeny types. Larvae were reared on either *Dicliptera canescens* or *Mikania micrantha* , and mean ± SEM survival rates are shown for each developmental stage panel (closed symbols with error bars). Light blue triangles, *D × D*; dark blue squares, *D × M*; dark green inverted triangles, *M × D*; light green circles, *M × M*. In each combination, the first letter denotes the female race and the second letter denotes the male race.

Larvae of neither host race in the first instar stage accepted leaves of the alternative host plant (*D* × *D* and *M* × *M*; Figure 5) and starved to death. Reciprocal F_1_ hybrids (*D* × *M* and *M* × *D*) survived on both host plants but had lower fitness compared to non‐hybrid offspring from the 2nd instar stage onward. The survivorship to adulthood of hybrid *MD* offspring was significantly lower than that of non‐hybrid offspring of *MM* on their maternal food plant *M. micrantha* (*M* × *D* vs. *M* × *M*, estimate = −2.16 ± 0.36, *p* < 0.0001; 0.603 ± 0.078 vs. 0.890 ± 0.035). Likewise, the survivorship of *D* × *M* larvae to adulthood was significantly lower than that of non‐hybrid offspring of *D* × *D* on their maternal food plant *D. canescens* (*D* × *M* vs. *D* × *D*, estimate = 2.09 ± 0.65, *p* = 0.0028; 0.178 ± 0.097 vs. 0.658 ± 0.076) (complete GLMM results are provided in Tables S9–S11). Hybrid survival to the 2nd instar (*D* × *M* and *M* × *D*) did not differ significantly (*D* × *M* vs. *M* × *D* in *D*, estimate = −1.13 ± 0.94, *p* = 0.228, 0.289 ± 0.198 vs. 0.559 ± 0.085; *D* × *M* vs. *M* × *D* in *M*, estimate = −1.92 ± 0.98, *p* = 0.101, 0.133 ± 0.133 vs. 0.656 ± 0.078; Table S10), although almost all *D* × *M* larvae failed to reach adulthood (*D* × *M* vs. *M* × *D* in *D*, estimate = −1.09 ± 0.64, *p* = 0.0876; *D* × *M* vs. *M* × *D* in *M*, estimate = −2.17 ± 0.75, *p* = 0.0074; Table S10).

### 3.7 Mate Choice in the Absence and Presence of Host Plants

**FIGURE 6:**
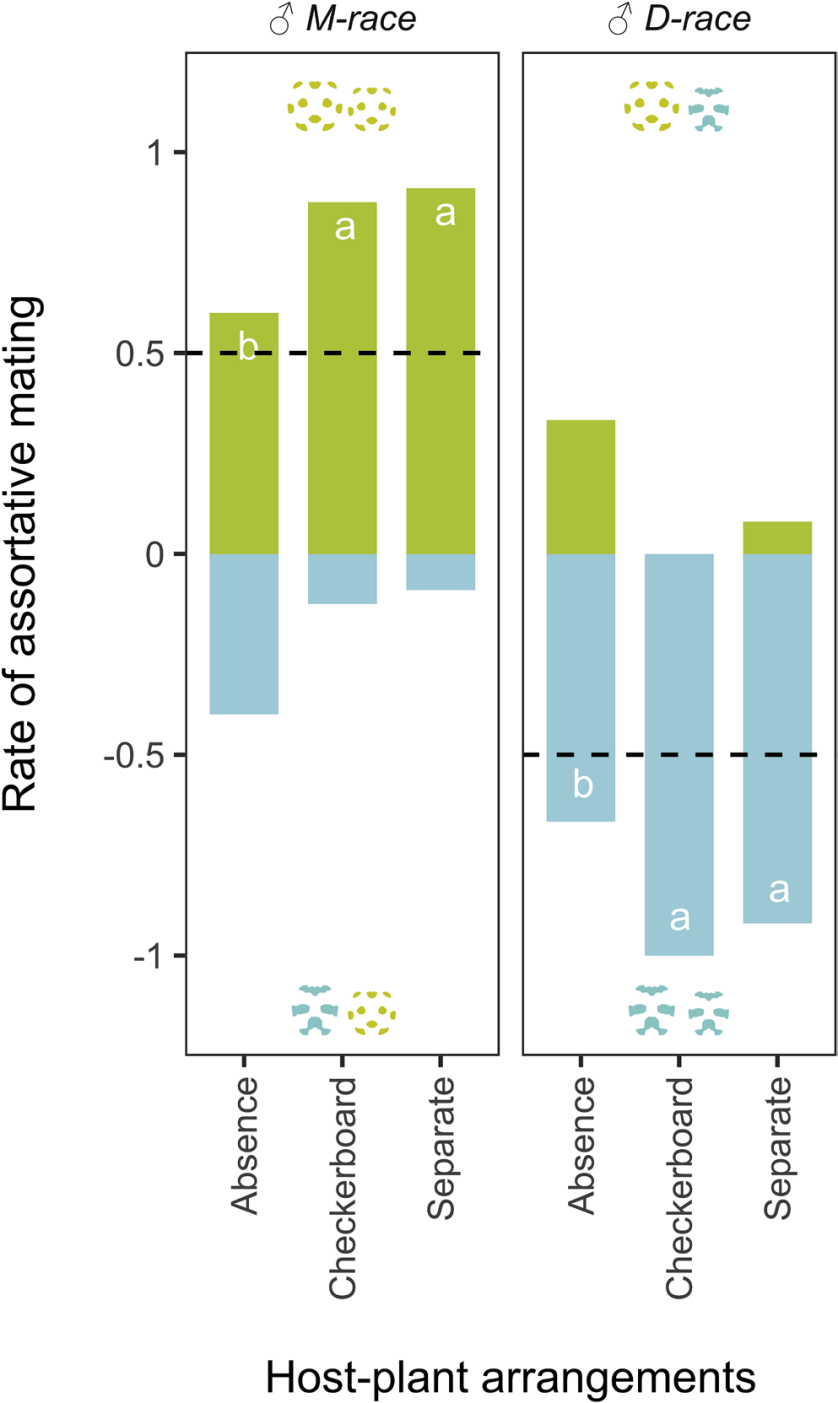
The rate of assortative mating on the total number of copulations for each host race of *Henosepilachna diekei* in the absence and presence of host plants. Mate choice in the absence of host plants was tested in the laboratory, while that in the presence of host plants was tested in field cages arranged in either a separate (parapatric) or checkerboard (sympatric) design. The rate of assortative mating (random mating 0.5) is indicated by a dashed line and signs indicate the direction of assortative mating.

Mating patterns differed markedly between conditions with and without host plants. In the absence of host plants (laboratory mate choice assay), males of both host races mated approximately at random with no significant assortative mating detected (Figure 6). In contrast, in the presence of host plants (field‐cage experiments), both host races exhibited a high degree of assortative mating, with only a small number of inter‐racial matings observed in both S‐cage and C‐cage (Figure 6). GLM analysis indicated a significant effect of host plant presence on assortative mating patterns (Wald χ^2^ = 6.7161, d.f. = 1, *p* = 0.0096; Table S12), whereas host plant arrangement (separate vs. checkerboard) did not significantly affect mating behaviour (Wald χ^2^ = 0.0293, d.f. = 1, *p* = 0.8641; Table S12).

### 3.8 Host Fidelity and Dispersal

**FIGURE 7:**
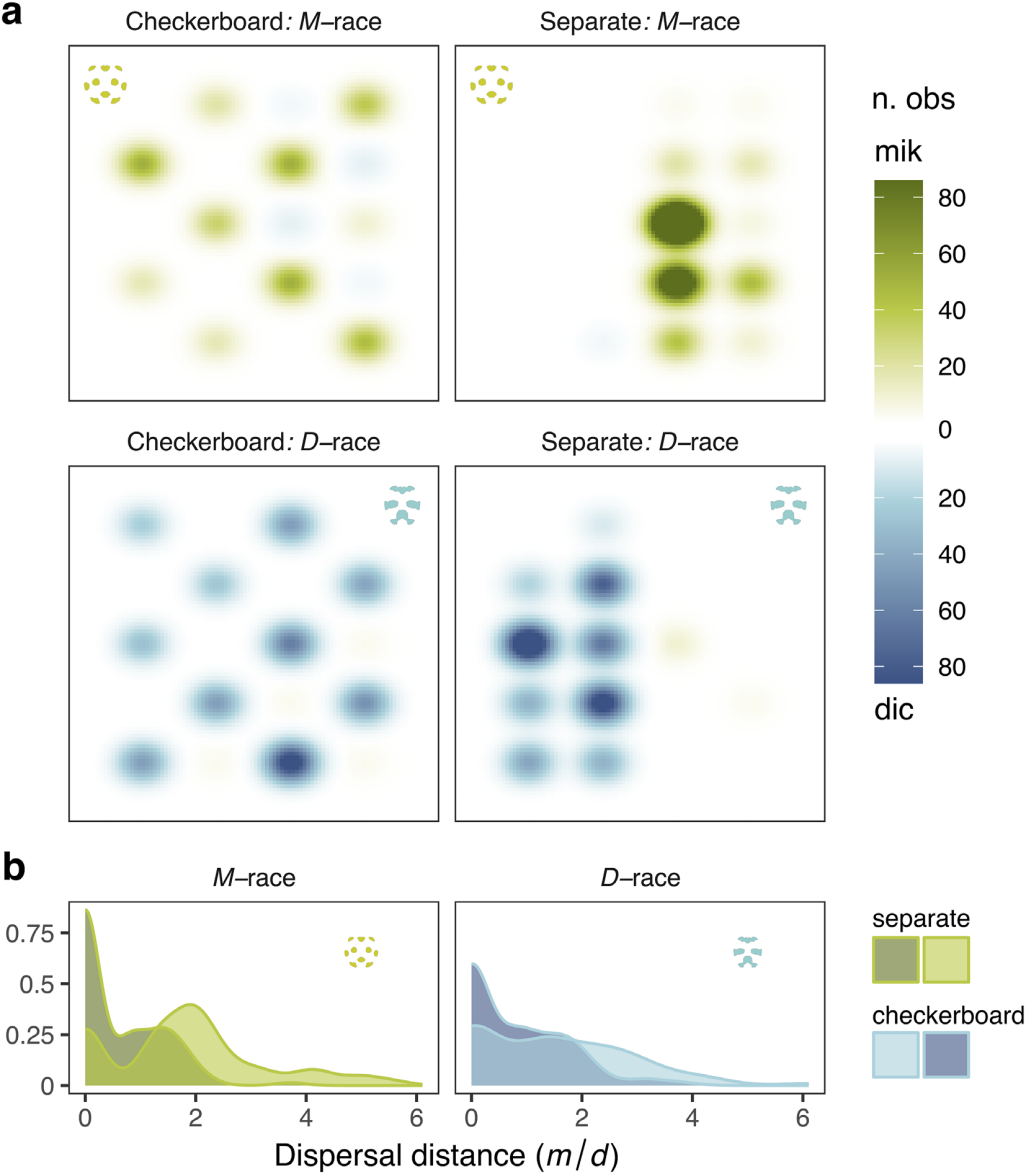
Host fidelity heatmap (a) and dispersal distance plot (b) of each host race of *Henosepilachna diekei* in field‐cage experiments. Each cage is visualised as two images based on beetle type and positioned under each other. Fidelity heatmaps are based on manual sighting records of individual beetles, with colour intensity indicating the number of sightings or observations (n. obs.) on *Mikania micrantha* (mik, green) or *Dicliptera canescens* (dic, blue). Dispersal distances are based on movement records of each individual beetles between observation periods. Darker colours represent the separate (S‐cage) treatment, and lighter colours represent the checkerboard (C‐cage) treatment. Details of the host plant arrangements are described in the main text.

The migration pattern shown in the heatmap of sighting events, illustrating the movement of beetles from both host races, was largely limited to their natal host plant even under the most sympatric patch scenario (Figure 7a; Table S13). In line with the sighting records, most adults of both sexes (85.9%) were sighted only on their natal host plant, and there were few instances of inter‐host migration or resting on the net. GLM analysis showed that the occurrence of beetles in the field cage was strongly affected by race (estimate = 6.83 ± 0.93, *p* < 0.0001), but not by sex or host plant arrangement (Table S13). The direction of migration was also significantly affected by race (estimate = 7.67 ± 0.93, *p* < 0.0001) and host plant arrangement (estimate = 1.43 ± 0.68, *p* = 0.0363), but not by sex (Table S13). Dispersal distances over 24 h varied from 0.5 m to 1.67 m (Figure 7b) and were significantly higher in C‐cage (estimate = −0.53 ± 0.25, *p* = 0.0339; Table S14). The frequency of intra‐host migration ranged from 0.41 m to 0.8 m, whereas inter‐host migration was quite low, ranging from 0 to 0.12 m (Table S13).

### 3.9 Strength of the Reproductive Isolation

**FIGURE 8:**
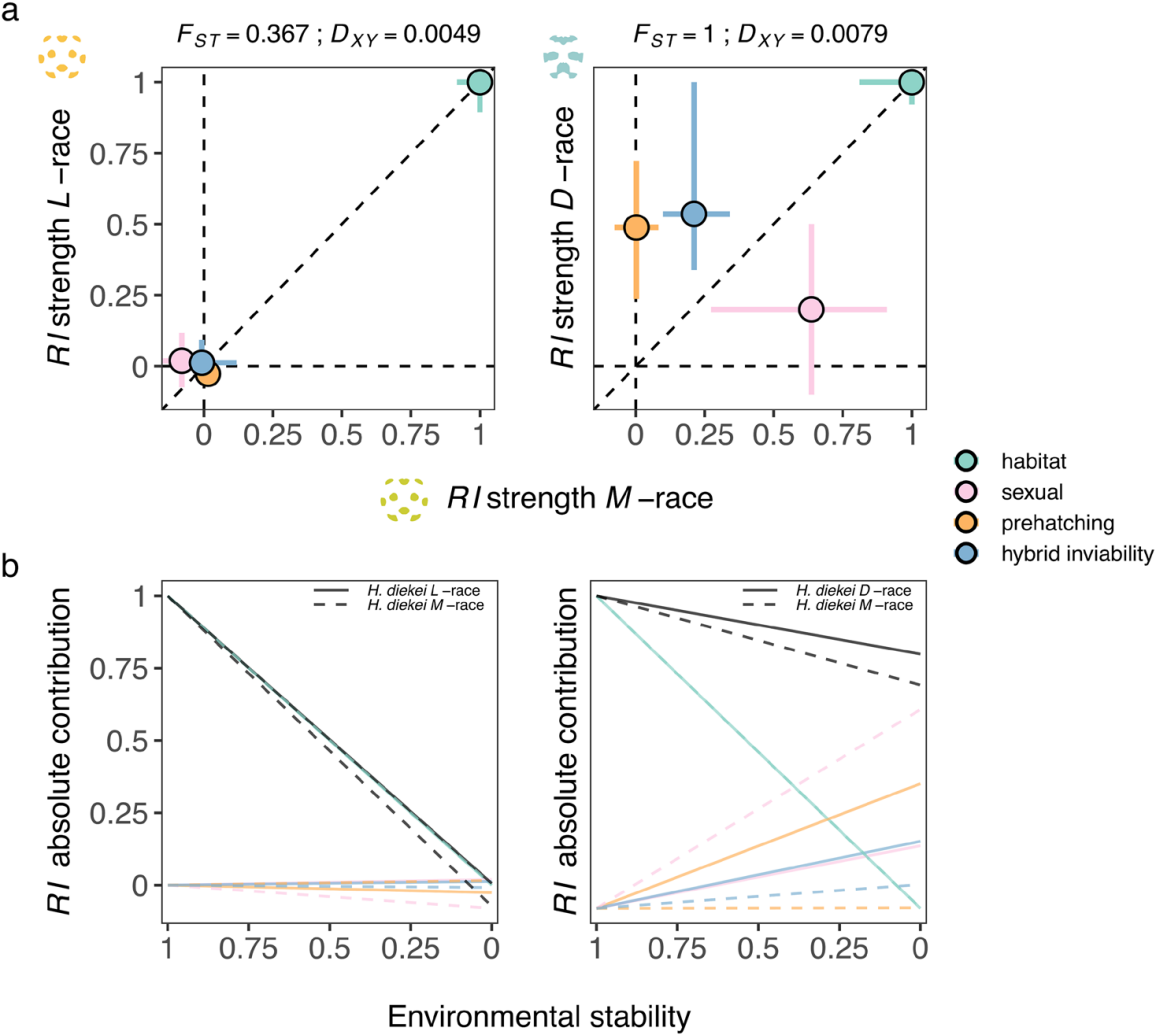
Strength and asymmetry of reproductive isolation (a) and the absolute contributions of each barrier under varying levels of environmental stability (b) at two levels of divergence in *Henosepilachna diekei*. *RI* values for each race represent directional estimates based on reciprocal crosses for mating and postmating barriers, and race‐specific estimates of encounter probability for habitat isolation. For example, *M*‐race values correspond to crosses involving *M*‐race females (*M* × *D* relative to *M* × *M*), and *D*‐race values correspond to crosses involving *D*‐race females (*D* × *M* relative to *D* × *D*). The left plot shows comparisons between *M‐* and *L‐*races, and the right plot shows comparisons between *M‐* and *D‐*races. In panel (a), each point represents the estimated strength of reproductive isolation (*RI*) for an individual barrier, with horizontal and vertical error bars indicating 95% confidence intervals obtained from 10,000 bootstrap replicates. Colours denote different reproductive barriers. In panel (b), solid lines represent the contributions of each barrier for the first race listed (*L‐* or *D‐*race), and dashed lines represent those for the *M‐*race. The black line indicates total *RI*. Environmental stability is shown on the x‐axis, where stability = 1 corresponds to no disturbance and stability = 0 corresponds to maximum disturbance.

Based on the results described above, we estimated the strength of each *RI* barrier and its absolute contribution across four distinct ecological scenarios (Figure 8). *RI* was calculated separately for each host race as directional estimates (maternal race first for mating and postmating barriers), and 95% confidence intervals (CI) were estimated where possible. Values in brackets indicate 95% confidence intervals.

Between the *M*‐ and *D*‐races, habitat isolation was complete in both races (*RI* = 1; *M*‐female [0.81–1]; *D*‐female [0.92–1]; Figure 8a). However, the strength of subsequent barriers varied and showed clear asymmetry. Sexual isolation was stronger in the *M*‐female direction (*RI* = 0.636 [0.273–0.909]) than in the *D*‐female direction (*RI* = 0.200 [−0.10–0.50]). Conversely, postmating barriers were stronger in the *D*‐female direction, including reduced egg hatchability (postmating‐prehatching, *RI* = 0.489 [0.237–0.723] vs. 0.002 [−0.077–0.082] in the *M*‐female direction) and F_1_ hybrid inviability (postmating‐posthatching, *RI* = 0.536 [0.339–1] vs. 0.211 [0.099–0.341]). However, we did not distinguish whether this F_1_ hybrid inviability represented an extrinsic or intrinsic postzygotic barrier.

Under baseline conditions, in the absence of ecological disturbance, total reproductive isolation was complete (${\mathrm{RI}}_{\text{total}}$ = 1), primarily attributed to habitat isolation alone. As the strength of habitat isolation decreased under simulated disturbance, the absolute contributions of later‐acting barriers increased. In the *M*‐female direction, sexual isolation became the dominant contributor under disturbance (*RI* = 0.318, 0.477, 0.636, for moderate, strong and extreme disturbance, respectively). Conversely, in the *D*‐female direction, the effect of other barriers was more evenly distributed, with a slightly greater emphasis on postmating barriers, particularly evident in the form of reduced egg hatchability (*RI =* 0.195, 0.293, 0.391, for moderate, strong and extreme disturbance, respectively) (Figure 8b).

**FIGURE 9:**
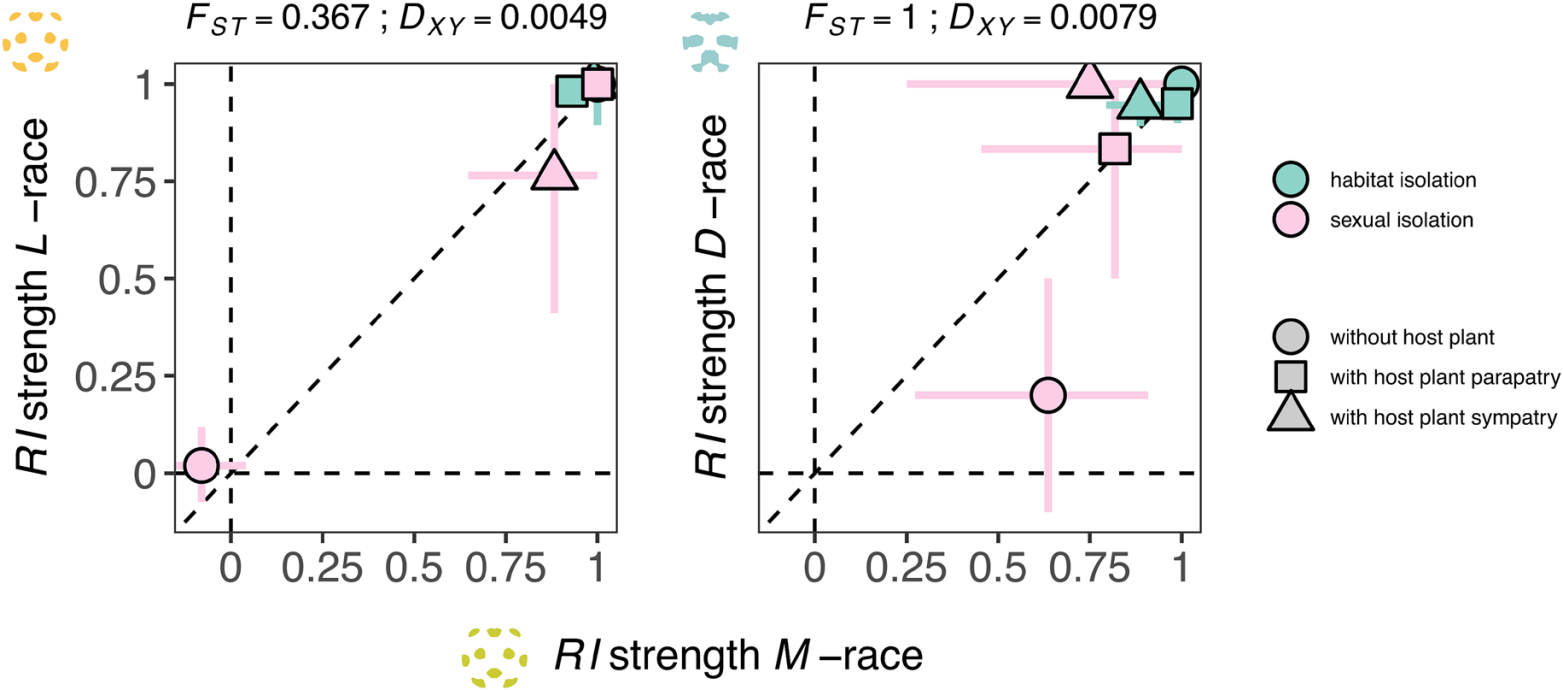
Effects of host‐plant context on habitat isolation and sexual isolation between host races of *Henosepilachna diekei*. Reproductive isolation (RI) estimates are shown for the *Mikania*–*Leucas* race pair (left) and the *Mikania*–*Dicliptera* race pair (right). The x‐axis shows *RI* strength for the *M*‐race, and the y‐axis shows *RI* strength for the *L*‐race or *D*‐race. Colours indicate barrier type. Shapes indicate experimental context. Error bars show 95% bootstrap confidence intervals. Dashed horizontal and vertical lines indicate *RI* = 0, and the diagonal dashed line indicates equal *RI* strength between reciprocal race directions. The presence of host plants strongly increased realised sexual isolation relative to laboratory estimates without host plants, especially in the *Mikania*–*Leucas* pair, where laboratory sexual isolation was weak but field‐cage sexual isolation was high. Genetic differentiation and absolute divergence are shown above each panel.

In contrast, between the *M*‐ and *L*‐races, habitat isolation was the only strong reproductive barrier (*RI* = 1; Figure 8a). All other barriers were weak and not clearly different from zero in either direction. Sexual isolation was close to zero (*M*‐female: *RI* = −0.08 [−0.21–0.04]; *L*‐female: *RI* = 0.02 [−0.07–0.12]), and postmating barriers were similarly minimal, with egg hatchability and hybrid inviability near zero or only weakly positive. However, when host plants were present in field‐cage experiments, realised sexual isolation increased sharply, apparently because strong host fidelity limited encounters between heterospecific pairs. This pattern was observed in both the *Mikania*–*Leucas* race pair (in sympatry, *M*‐female: *RI* = 1 [1–1]; *L*‐female: *RI* = 1 [1–1]) and the *Mikania*–*Dicliptera* race pair (in sympatry, *M*‐female: *RI* = 0.88 [0.79–0.96]; *D*‐female: *RI* = 0.95 [0.89–0.99]) (Figure 9). All *RI* estimates reported using the standardised RIO coding framework are provided in Table S15.

## 4 Discussion

### 4.1 The Evolution of Reproductive Isolation Beyond a Strong First Ecological Barrier

This study examined whether additional reproductive barriers can accumulate after the evolution of strong host‐associated habitat isolation in the phytophagous ladybird beetle *Henosepilachna diekei*. We found that the *Dicliptera–Mikania* race pair was more genetically differentiated than the previously studied *Mikania*–*Leucas* race pair in West Java, and that it showed not only strong habitat isolation but also additional later‐acting barriers. We identified three additional reproductive barriers to habitat isolation (and immigrant inviability), that is, sexual isolation (but see explanation below), reduced egg hatchability and F_1_ hybrid inviability. Meanwhile, the previously studied *Mikania*–*Leucas* race pair showed strong habitat isolation but no evidence for additional sexual or postmating barriers. This comparison suggests that even when habitat isolation is very strong, the accumulation of later barriers is still possible, although their contribution to total reproductive isolation is likely to be effective mainly in the presence of host‐associated habitat differences. Thus, these later‐acting barriers may evolve at the early stages of speciation, even during divergence among host races.

There are two broad predictions for the evolution of later barriers under strong habitat isolation. First, strong habitat isolation reduces inter‐racial encounters and hybridisation, which may limit the opportunity for reinforcement. In the absence of selection against maladaptive hybrids or residual gene flow between divergent populations, coupling among barrier loci may be difficult to evolve, as it often requires some degree of contact (Servedio and Noor 2003; Butlin and Smadja 2018; Rosser et al. 2019; Kulmuni et al. 2020). Consequently, under this expectation, additional sexual or postmating barriers might remain weak and evolve more slowly. On the other hand, the same reduction in inter‐racial contact also reduces gene flow, which can allow behavioural, physiological, or genetic divergence to accumulate through drift and the buildup of incompatibilities in geographically isolated or weakly connected populations (Schluter 2009; Seehausen et al. 2014; Kulmuni et al. 2020). Under this second expectation, later barriers may still evolve after habitat isolation has become strong, not necessarily because they are directly favoured by frequent hybridisation, but because the first barrier allows host races to continue diverging with little genetic exchange.

Our results are more consistent with the second possibility. Several ecological aspects may explain the emergence of these additional barriers in the host races of *H. diekei*. The *D*‐race is confined to the southern Bandung mountains, particularly Mount Patuha (Matsubayashi et al. 2015, 2016), and showed larger genetic ND2‐based mitochondrial differentiation from the *M*‐race than the previously studied *Mikania*–*Leucas* race comparison. This pattern is consistent with, but does not by itself demonstrate, a history of stronger geographic isolation from other host races. Consequently, contemporary gene flow between these races may be limited, although this inference requires confirmation using genome‐wide markers. If such limited migration is confirmed, the development of *RI* via reinforcement would be less likely under this micro‐allopatric scenario (e.g., Rosser et al. 2019), as predicted by many theoretical models (Uyeda et al. 2009; Kyogoku and Kokko 2020; Yukilevich et al. 2024). Instead, reduced migration could facilitate the accumulation of genetic incompatibilities. Sexual isolation is only likely to evolve towards targeting a neutral discriminant trait over a trait providing a fitness advantage when the risk of choosing the wrong mate is too high, indicating that adaptation and mate choice may not act synergistically (McPeek and Gavrilets 2006; Yamaguchi and Iwasa 2013; Blanckaert and Sousa 2025).

This contrast implies that the evolution of such barriers is highly contingent on the specific speciation trajectory. While postmating barriers have been reported between host‐associated populations in other systems (e.g., Egan et al. 2011; McBride and Singer 2010), they have not been identified among species pairs of another closely related phytophagous ladybird beetles in *Henosepilachna vigintioctomaculata* complex (Katakura et al. 1981, 1989; Katakura and Hosogai 1994; Katakura 1997; Kuwajima et al. 2010). Therefore, the *Dicliptera–Mikania* race pair suggests that later barriers do not replace the importance of the initial host‐associated barrier. Rather, the emergence of new barriers is more likely to strengthen and stabilise existing barriers evolved earlier (i.e., habitat isolation) via coupling of barriers (Butlin and Smadja 2018; Dopman et al. 2024).

### 4.2 Possible Mechanisms Underlying Later‐Acting Barriers

Although plant‐derived chemicals have been implicated in sexual isolation via chemosensory system divergence (i.e., pheromones or cuticular hydrocarbons (CHCs)) in many phytophagous insects (Symonds and Elgar 2008; Lassance and Löfstedt 2013; Schwander et al. 2013; Chung and Carroll 2015; Van Schooten et al. 2020; Hood et al. 2021; Lackey et al. 2023), host‐associated premating isolation need not require genetically fixed divergence in sensory structures. Developmental host plant or diet can also induce rapid plastic changes in mating cues, mate preference, or sexual behaviour, thereby generating sexual isolation without underlying genetic divergence in chemosensory systems (Hubakk et al. 2024; Jarrett and Miller 2024). Comparative studies in epilachnine beetles suggest that this mechanism may not be an essential component in their speciation (Katakura 1997; Nakano and Abbas 1994; Matsubayashi and Katakura 2007). In which, in extreme cases, distantly related *Henosepilachna* species have been observed to form heterospecific mating pairs (Nakano and Abbas 1994). While we did detect reduced mating attempts and success between the *M*‐ and *D*‐races, we argue this pattern likely reflects general behavioural differences rather than divergence in mating cues. Specifically, *M*‐race individuals were noticeably more active in experimental arenas, which likely enhanced their chances of encountering and engaging with mates. Conversely, *D*‐race beetles exhibited relatively passive behaviour, even in conspecific pairings, potentially due to a reduced mate‐search drive. These behavioural tendencies are a more plausible explanation for the observed sexual isolation than divergence in chemosensory systems.

By contrast, postmating barriers may evolve relatively rapidly in allopatric populations such as those studied here. Garlovsky et al. (2023) suggest that postmating barriers such as reduced egg hatchability (regardless of gametic isolation or early embryonic inviability) can evolve quickly, possibly because they require only a few mutational changes (Vacquier and Swanson 2011). Since genes involved in fertilisation tend to be highly accommodative between different sexes of the same species, minor divergences between taxa can disrupt reproductive compatibility (Dean et al. 2009; Finseth et al. 2014; Kasimatis and Phillips 2018; Garlovsky et al. 2022). Although we did not determine the precise mechanism in our study system, unfertilised eggs and early hybrid mortality are likely contributors to the low hatching rates, consistent with interspecific crosses reported between group A and B *H. vigintioctomaculata* and *H. pustulosa* (Katakura and Sobu 1986; Kohyama et al. 2016).

Similarly, ecological (extrinsic) hybrid inviability is predicted to emerge early during speciation (Seehausen et al. 2014). Hybrid fitness often depends heavily on environmental context (e.g., Via et al. 2000; Forister 2005; Craig et al. 2007; Ohshima 2008; McBride and Singer 2010; Zhang et al. 2021), and this has been demonstrated in *H. niponica* and *H. yasutomii* pair (Kuwajima et al. 2010) as well as in the *M*‐race and *L*‐race pair of *H. diekei* (unpublished data). In these cases, selection against intermediate phenotypes may arise from reduced host‐search efficiency (Tosh et al. 2009). However, the strength of such barriers is variable: weak hybrid inviability has been reported even between more genetically distant species pairs of the phytophagous ladybird beetles such as *H. vigintioctomaculata* and *H. pustulosa* (Katakura 1997; Matsubayashi and Katakura 2009), whereas we observed much stronger isolation between the *M*‐ and *D*‐race of *H. diekei*. Negative epistasis for fitness between opposite‐ancestry alleles could thus be independent of ecological niche (intrinsic hybrid inviability) and could be explained by the different mechanisms giving rise to this barrier (Reifová et al. 2023). For instance, barriers arising due to underdominance of chromosomal rearrangements could lead to stronger barriers compared to DMIs arising from a few loci (e.g., Luo et al. 2018), indicating a possible snowball effect of genomic incompatibilities (Satokangas et al. 2020).

### 4.3 Dynamics of the Evolution of Reproductive Barriers in Phytophagous Ladybird Beetles

Although our findings, along with existing comparative data, do not yet permit definitive conclusions about the broader dynamics of reproductive isolation in phytophagous ladybird beetles, they provide useful preliminary insights when viewed from a speciation continuum perspective (Stankowski and Ravinet 2021), which involves placing divergent populations (within or between taxa) according to their current level of *RI*to infer the rate and order of evolution of different forms of *RI* (Coyne and Orr 1989, 1997; Barton and de Cara 2009; Nosil et al. 2009; Smadja and Butlin 2011; Flaxman et al. 2013; Roux et al. 2016; Mérot et al. 2017; Servedio and Hermisson 2019; Matute and Cooper 2021). During the transition from populations to species, divergence is likely to be heterogenous rather than uniform and gradual (Nosil et al. 2024; Jarrett et al. 2025). For instance, in phytophagous ladybird beetles, habitat isolation appears to be accumulated non‐linearly early in divergence, whereas other barriers may follow a more linear progression with increasing population or species differentiation. Abrupt shifts from weak to strong *RI* may be driven by changes in geographical distribution, demographic events such as population bottlenecks, the origin of large‐effect mutations, or the non‐linear accumulation of genetic incompatibilities (Butlin et al. 2008; Nosil et al. 2024). Muschick et al. (2020) proposed that shifts to more distantly related hosts are rare but tend to result in greater *RI*, although the host divergence considered in their study occurred at a much deeper phylogenetic scale than that among the host plants examined here. Nevertheless, for specialist phytophagous insects, shifts among different angiosperm families may still represent substantial ecological transitions. This may explain why, although host plant specialisation (i.e., habitat isolation) consistently emerges as the first and primary barrier in the early stages of speciation in phytophagous ladybird beetles (as repeatedly demonstrated in Katakura 1997; Matsubayashi and Katakura 2009; Matsubayashi et al. 2010, 2011; Matsubayashi, Kahono, and Katakura 2013), additional postmating barriers, such as those identified in this study, evolve only in some populations or species pairs. Future comparative studies should examine a wider range of population pairs that span a range of degrees of differentiation, among and within geographically distributed host races (e.g., Powell et al. 2013; Gompert et al. 2014; Soria‐Carrasco et al. 2014; Riesch et al. 2017; Nosil et al. 2017). Such work could clarify whether greater divergence enhances the likelihood that other inherited factors accelerate the rate of evolution of *RI* between populations, while also shedding light on the relative influence of genetic versus environmental drivers and disentangling the roles of ecological and mutation‐order speciation across the speciation continuum in phytophagous ladybird beetles (e.g., Mérot et al. 2017; Anderson and Weir 2022; de Carvalho et al. 2024).

## Author Contributions

**Arif Maulana:** conceptualization (lead), data curation (lead), formal analysis (lead), funding acquisition (lead), investigation (lead), resources (lead), visualization (lead), writing – original draft (lead), writing – review and editing (lead). **Kei W. Matsubayashi:** formal analysis (equal), investigation (equal), visualization (equal), writing – review and editing (equal). **Naoyuki Fujiyama:** formal analysis (equal), writing – review and editing (equal). **Tri Atmowidi:** conceptualization (equal), resources (equal), supervision (equal), writing – review and editing (supporting). **Sih Kahono:** conceptualization (equal), resources (equal), supervision (equal), writing – review and editing (supporting).

## Funding

This work was supported by Wellcome Trust (Grant 220540/Z/20/A).

## Conflicts of Interest

The authors declare no conflicts of interest.

## Supporting information


**Table S1:** Details of the observation sites of *Henosepilachna diekei* and its host plants.
**Table S2:** Results of adult host preference in the two host races of *Henosepilachna diekei* collected from 5 sites (5 populations) in the vicinities of southern Bandung, West Java, Indonesia.
**Table S3:** Results of the generalised linear model (GLM) testing adult host preference for each population of *Henosepilachna diekei*.
**Table S4:** Results of the generalised linear model (GLM) testing mating attempts in each race of *Henosepilachna diekei*.
**Table S5:** Results of the generalised linear model (GLM) testing mating success in each race of *Henosepilachna diekei*.
**Table S6:** Results of the generalised linear model (GLM) testing the number of eggs produced per batch by each mating combination between host races of *Henosepilachna diekei*.
**Table S7:** Results of the generalised linear model (GLM) testing the number of eggs hatched per batch by each mating combination between host races of *Henosepilachna diekei*.
**Table S8:** Results of the generalised linear model (GLM) testing hatching duration per egg batch from each mating combination between host races of *Henosepilachna diekei*.
**Table S9:** Results of the generalised linear model (GLM) testing larval performance of parental and hybrid types of host races of *Henosepilachna diekei* across hosts and developmental stages (1).
**Table S10:** Results of the generalised linear model (GLM) testing larval performance of parental and hybrid types of host races of *Henosepilachna diekei* across hosts and developmental stages (2).
**Table S11:** Results of the generalised linear model (GLM) testing larval performance of parental and hybrid types of host races of *Henosepilachna diekei* across hosts and developmental stages (3).
**Table S12:** Results of the generalised linear model (GLM) testing host‐associated assortative mating in host races of *Henosepilachna diekei*.
**Table S13:** Details of migration and host fidelity data of host races of *Henosepilachna diekei* in the field cages.
**Table S14:** Results of the generalised linear model (GLM) testing host fidelity and dispersal of host races of *Henosepilachna diekei* in the field cage experiments.
**Table S15:** Empirical reproductive isolation (*RI*) estimates classified according to the reproductive isolation ontology (RIO) framework.

## Data Availability

The data and computer scripts used in the analysis are publicly accessible at https://github.com/diekei/2026_EE_diekei_dicliptera_speciation and are permanently archived in Zenodo at https://doi.org/10.5281/zenodo.20767805. The ND2 haplotype sequence generated in this study has been deposited in the DNA Data Bank of Japan (DDBJ) under accession number LC940254.
