# Supplementary material for: The Evolution of Reproductive Isolation Beyond a Strong First Barrier in Speciation Between Micro‐Allopatric Host Races of a Phytophagous Ladybird Beetle, Henosepilachna diekei
Source: Ecol Evol. 2026 Jul 17;16(7):e74046. doi: 10.1002/ece3.74046 (PMC13378412; doi:10.1002/ece3.74046)
Supplement: Supplementary file 1 — Table S1: Details of the observation sites of Henosepilachna diekei and its host plants. Table S2: Results of adult host preference in the two host races of Henosepilachna diekei collected from 5 sites (5 populations) in the vicinities of southern Bandung, West Java, Indonesia. Table S3: Results of the generalised linear model (GLM) testing adult host preference for each population of Henosepilachna diekei. Table S4: Results of the generalised linear model (GLM) testing mating attempts in each race of Henosepilachna diekei. Table S5: Results of the generalised linear model (GLM) testing mating success in each race of Henosepilachna diekei. Table S6: Results of the generalised linear model (GLM) testing the number of eggs produced per batch by each mating combination between host races of Henosepilachna diekei. Table S7: Results of the generalised linear model (GLM) testing the number of eggs hatched per batch by each mating combination between host races of Henosepilachna diekei. Table S8: Results of the generalised linear model (GLM) testing hatching duration per egg batch from each mating combination between host races of Henosepilachna diekei. Table S9: Results of the generalised linear model (GLM) testing larval performance of parental and hybrid types of host races of Henosepilachna diekei across hosts and developmental stages (1). Table S10: Results of the generalised linear model (GLM) testing larval performance of parental and hybrid types of host races of Henosepilachna diekei across hosts and developmental stages (2). Table S11: Results of the generalised linear model (GLM) testing larval performance of parental and hybrid types of host races of Henosepilachna diekei across hosts and developmental stages (3). Table S12: Results of the generalised linear model (GLM) testing host‐associated assortative mating in host races of Henosepilachna diekei. Table S13: Details of migration and host fidelity data of host races of Henosepilachna diekei in the field cage [file ECE3-16-e74046-s001.docx]

**Table S1.** Details of the observation sites of *Henosepilachna diekei* and its host plants.

| ID | Site | Latitude (d.d) | Longitude (d.d) | Altitude (m) | Race | Host^1)^ |
| --- | --- | --- | --- | --- | --- | --- |
| 1 | Dewata | -7.203103749 | 107.4632565 | 1497 | *-* | *-* |
| 2 | Rancabolang | -7.172703304 | 107.4375434 | 1827 | *-* | *-* |
| 3 | Patenggang Lake | -7.170945569 | 107.3598013 | 1621 | *-* | *-* |
| 4 | Mount Pasir Cadasdayang | -7.122900812 | 107.3725239 | 1724 | *-* | *-* |
| 5 | Rancabali | -7.150656285 | 107.3656939 | 1659 | *D* | *D* |
| 6 | Rancabali Tea Plantation | -7.147408660 | 107.3777469 | 1761 | *-* | *-* |
| 7 | Mount Pasir Cadaspanjang - Cimanggu | -7.144604654 | 107.3877241 | 1758 | *D* | *D* |
| 8 | Rancaupas | -7.141064074 | 107.3917418 | 1742 | *-* | *M* |
| 9 | White Crater –  Sunan Ibu | -7.168468000 | 107.4044980 | 2245 | *-* | *-* |
| 10 | Mount Patuha | -7.143759268 | 107.3977014 | 1760 | *D* | *D* |
| 11 | Mount Tikukur | -7.133024529 | 107.3976428 | 1735 | *D* | *D* |
| 12 | Patuha Punceling | -7.141231477 | 107.4152453 | 1636 | *-* | *-* |
| 13 | Punceling | -7.134049874 | 107.4101562 | 1623 | *D* | *D* |
| 14 | Patuha Resort | -7.128458603 | 107.4102232 | 1503 | *-* | *M* |
| 15 | Rancabali Forest | -7.119100757 | 107.4142408 | 1564 | *-* | *M* |
| 16 | Mount Tambakruyung | -7.101581998 | 107.4218577 | 1348 | *M* | *M* |
| 17 | Cilember | -7.085594980 | 107.4187607 | 1382 | *-* | *-* |
| 18 | Padalarang | -6.813765448 | 107.4721289 | 605 | *M* | *M* |
| 19 | Bandung | -6.848551860 | 107.6443535 | 992 | *M* | *M* |
| 20 | Cimahi Waterfall | -6.805930972 | 107.5752494 | 1187 | *-* | *M* |
| 21 | Layung Waterfall | -6.785574727 | 107.5816776 | 1485 | *-* | *-* |
| 22 | Mount Tangkubanparahu | -6.771646769 | 107.6327022 | 1546 | *-* | *-* |

d.d = decimal degrees; m = meter

^1)^ *D = Dicliptera canescens*, *M = Mikania micrantha*

**Table S2.** Results of adult host preference in the two host races of *Henosepilachna diekei* collected from 5 sites (5 populations) in the vicinity of southern Bandung, West Java, Indonesia.

|  | Locality | Sex | *N* | Fed only on *Mikania* | | Fed only on *Dicliptera* | | No. of null choice |
| --- | --- | --- | --- | --- | --- | --- | --- | --- |
| Natal host |  |  |  | *N* | Leaf area  consumed (mm^2^) ± SE | *N* | Leaf area  consumed (mm^2^) ± SE |  |
| *M. micrantha* | Bandung | female | 4 | 4 | not measured | 0 | 0 | 0 |
| *M. micrantha* | Bandung | male | 2 | 2 |  | 0 |  | 0 |
| *M. micrantha* | Padalarang | female | 7 | 7 | 76.92 ± 17.45 | 0 | 0 | 0 |
| *M. micrantha* | Padalarang | male | 10 | 10 |  | 0 |  | 0 |
| *M. cordata* | Tambakruyung | female | 6 | 6 | 105.63 ± 28.03 | 0 | 0 | 0 |
| *M. cordata* | Tambakruyung | male | 8 | 6 |  | 0 |  | 2 |
| *D.* *canescens* | Patuha | female | 13 | 0 | 0 | 12 | 55.75 ± 7.80 | 1 |
| *D. canescens* | Patuha | male | 29 | 0 |  | 27 |  | 2 |
| *D. canescens* | Rancabali | female | 24 | 0 | 0 | 20 | 69.47 ± 8.44 | 4 |
| *D. canescens* | Rancabali | male | 24 | 0 |  | 20 |  | 4 |

**Table S3.** Results of the generalised linear model (GLM) testing adult host preference for each population of *Henosepilachna diekei*. Significant P-values (α = 0.05) are highlighted in bold.

| Comparison | Estimate | SE | t value | Pr (> \| t \|) |
| --- | --- | --- | --- | --- |
| Factors determining the food preference | | | | |
| Intercept | -27.6983 | 0.4644 | -59.639 | **<0.0001** |
| Race (mrace) | 54.8502 | 0.6714 | 81.694 | **<0.0001** |
| Sex (male) | -0.1872 | 0.6267 | -0.299 | 0.768 |
| Race (mrace) × Sex (male) | 0.1961 | 0.9230 | 0.213 | 0.834 |
| Factors determining the food preference among *M*-race populations | | | | |
| Intercept | 26.3653 | 1.3381 | 19.704 | **<0.0001** |
| Population (pdl) | -0.3203 | 1.5916 | -0.201 | 0.847 |
| Population (tbr) | -0.2058 | 1.6620 | -0.124 | 0.905 |
| Sex (male) | -0.4629 | 2.0111 | -0.230 | 0.826 |
| Pop. (pdl) × Sex (male) | 0.6499 | 2.3268 | 0.279 | 0.789 |
| Pop. (tbr) × Sex (male) | 0.4650 | 2.4474 | 0.190 | 0.856 |
| Factors determining the food preference among *D*-race populations | | | | |
| Intercept | -26.3647 | 0.7676 | -34.345 | **<0.0001** |
| Population (ran) | -0.7424 | 1.1542 | -0.643 | 0.541 |
| Sex (male) | -0.3801 | 0.9861 | -0.386 | 0.711 |
| Pop. (ran) × Sex (male) | 0.3801 | 1.5678 | 0.242 | 0.815 |

mrace = *M*-race; pdl *=* Padalarang; tbr *=* Tambakruyung; ran = Rancabali

Significance of the fixed factors included in the generalised linear model testing adult host preference for each population of *H. diekei*. Significant P-values (α = 0.05) are highlighted in bold.

| Comparison | Factor | d.f. | Wald χ^2^ | P |
| --- | --- | --- | --- | --- |
| Factors determining the food preference | | | | |
|  | Race | 1 | 14228.0723 | **<0.0001** |
|  | Sex | 1 | 0.0442 | 0.8335 |
|  | Race × Sex | 1 | 0.0452 | 0.8317 |
| Factors determining the food preference among *M*-race populations | | | | |
|  | Population | 2 | 0.0003 | 0.9998 |
|  | Sex | 1 | 0.0004 | 0.9849 |
|  | Population × Sex | 2 | 0.0782 | 0.9617 |
| Factors determining the food preference among *D*-race populations | | | | |
|  | Population | 1 | 0.4716 | 0.4923 |
|  | Sex | 1 | 0.0898 | 0.7644 |
|  | Population × Sex | 1 | 0.0588 | 0.8084 |

**Table S4.** Results of the generalised linear model (GLM) testing mating attempts in each race of *Henosepilachna diekei*. Significant P-values (α = 0.05) are highlighted in bold.

| Comparison | Estimate | SE | z value | Pr (> \| z \|) |
| --- | --- | --- | --- | --- |
| Factors determining the mating attempt | | | | |
| Intercept | 0.5108 | 0.3266 | 1.564 | 0.1178 |
| Female (m) | -0.5108 | 0.5538 | -0.922 | 0.3563 |
| Male (m) | -0.8131 | 0.4571 | -1.779 | 0.0753 |
| Female (m) × Male (m) | 3.7575 | 1.2090 | 3.108 | **0.0019** |
| Pairwise comparison | | | | |
| ***D*** × *D –* ***D*** × *M* | 0.8131 | 0.4571 | 1.779 | 0.2259 |
| *D* × ***D*** *–* *M* × ***D*** | 0.5108 | 0.5538 | 0.922 | 0.7126 |
| *D* × *D –* *M* × *M* | -2.4336 | 1.0767 | -2.260 | 0.0952 |
| *D* × *M –* *M* × *D* | -0.3023 | 0.5498 | -0.550 | 0.7126 |
| *D* × ***M*** *–* *M* × ***M*** | -3.2467 | 1.0747 | -3.021 | **0.0151** |
| ***M*** × *D –* ***M*** × *M* | -2.9444 | 1.1192 | -2.631 | **0.0426** |

m = *M*-race

Significance of the fixed factors included in the generalised linear model testing mating attempts of each race of *H. diekei*. Significant P-values (α = 0.05) are highlighted in bold.

| Comparison | Factor | d.f. | Wald χ^2^ | P |
| --- | --- | --- | --- | --- |
| Factors determining the mating attempt | | | | |
|  | Female | 1 | 0.3179 | 0.5729 |
|  | Male | 1 | 0.4250 | 0.5145 |
|  | Male × Female | 1 | 9.6601 | **0.0019** |

**Table S5.** Results of the generalised linear model (GLM) testing mating success in each race of *Henosepilachna diekei*. Significant P-values (α = 0.05) are highlighted in bold.

| Comparison | Estimate | SE | z value | Pr (> \| z \|) |
| --- | --- | --- | --- | --- |
| Factors determining the mating success | | | | |
| Intercept | 0.4055 | 0.3227 | 1.256 | 0.2090 |
| Female (m) | -1.7918 | 0.6455 | -2.776 | **0.0055** |
| Male (m) | -0.8109 | 0.4564 | -1.777 | 0.0756 |
| Female (m) × Male (m) | 4.3944 | 1.0375 | 4.236 | **<0.0001** |
| Pairwise comparison | | | | |
| ***D*** × *D –* ***D*** × *M* | 0.8109 | 0.4564 | 1.777 | 0.4880 |
| *D* × ***D*** *–* *M* × ***D*** | 1.7918 | 0.6455 | 2.776 | **0.0220** |
| *D* × *D –* *M* × *M* | -1.7918 | 0.8122 | -2.206 | 0.0822 |
| *D* × *M –* *M* × *D* | 0.9808 | 0.6455 | 1.519 | 0.1513 |
| *D* × ***M*** *–* *M* × ***M*** | -2.6027 | 0.8122 | -3.204 | **0.0068** |
| ***M*** × *D –* ***M*** × *M* | -3.5835 | 0.9317 | -3.846 | **0.0007** |

m = *M*-race

Significance of the fixed factors included in the generalised linear model testing mating success in each race of *H. diekei*. Significant P-values (α = 0.05) are highlighted in bold.

| Comparison | Factor | d.f. | Wald χ^2^ | P |
| --- | --- | --- | --- | --- |
| Factors determining the mating success | | | | |
|  | Male | 1 | 0.0093 | 0.9230 |
|  | Female | 1 | 0.0322 | 0.8576 |
|  | Male × Female | 1 | 17.9407 | **<0.0001** |

**Table S6.** Results of the generalised linear model (GLM) testing the number of eggs produced per batch by each mating combination between host races of *Henosepilachna diekei*. Significant P-values (α = 0.05) are highlighted in bold.

| Comparison | Estimate | SE | z value | Pr (> \| z \|) |
| --- | --- | --- | --- | --- |
| Factors determining the number of eggs produced per batch | | | | |
| Intercept | 2.3195 | 0.0690 | 33.615 | **<0.0001** |
| Female (m) | 0.4071 | 0.0918 | 4.436 | **<0.0001** |
| Male (m) | -0.0786 | 0.0993 | -0.791 | 0.4290 |
| Female (m) × Male (m) | 0.0956 | 0.1252 | 0.764 | 0.4450 |
| Pairwise comparison | | | | |
| ***D*** × *D –* ***D*** × *M* | 0.0786 | 0.0993 | 0.791 | 0.8570 |
| *D* × ***D*** *–* *M* × ***D*** | -0.4071 | 0.0918 | -4.436 | **<0.0001** |
| *D* × *D –* *M* × *M* | -0.4241 | 0.0828 | -5.122 | **<0.0001** |
| *D* × *M –* *M* × *D* | -0.4857 | 0.0939 | -5.173 | **<0.0001** |
| *D* × ***M*** *–* *M* × ***M*** | -0.5027 | 0.0851 | -5.905 | **<0.0001** |
| ***M*** × *D –* ***M*** × *M* | -0.0170 | 0.0762 | -0.223 | 0.8570 |

m = *M*-race

Significance of the fixed factors included in the generalised linear model testing the number of eggs produced per batch by each mating combination between host races of *H. diekei*. Significant P-values (α = 0.05) are highlighted in bold.

| Comparison | Factor | d.f. | Wald χ^2^ | P |
| --- | --- | --- | --- | --- |
| Factors determining the number of eggs produced per batch | | | | |
|  | Female | 1 | 53.9294 | **<0.0001** |
|  | Male | 1 | 0.0926 | 0.7609 |
|  | Female × Male | 1 | 0.5835 | 0.4450 |

**Table S7.** Results of the generalised linear model (GLM) testing the number of eggs hatched per batch by each mating combination between host races of *Henosepilachna diekei*. Significant P-values (α = 0.05) are highlighted in bold.

| Comparison | Estimate | SE | z value | Pr (> \| z \|) |
| --- | --- | --- | --- | --- |
| Factors determining the number of eggs hatched per batch | | | | |
| Intercept | 1.1710 | 0.1615 | 7.251 | **<0.0001** |
| Female (m) | 0.7090 | 0.1876 | 3.779 | **0.0002** |
| Male type (m) | -1.0591 | 0.2466 | -4.295 | **<0.0001** |
| Female (m) × Male (m) | 1.0850 | 0.2777 | 3.907 | **<0.0001** |
| Pairwise comparison | | | | |
| ***D*** × *D –* ***D*** × *M* | 1.0591 | 0.2466 | 4.295 | **<0.0001** |
| *D* × ***D*** *–* *M* × ***D*** | -0.7090 | 0.1876 | -3.779 | **0.0003** |
| *D* × *D –* *M* × *M* | -0.7349 | 0.1728 | -4.253 | **<0.0001** |
| *D* × *M –* *M* × *D* | -1.7681 | 0.2211 | -7.996 | **<0.0001** |
| *D* × ***M*** *–* *M* × ***M*** | -1.7939 | 0.2095 | -8.565 | **<0.0001** |
| ***M*** × *D –* ***M*** × *M* | -0.0259 | 0.1266 | -0.205 | 0.8379 |
| Pairwise comparison (considering rate) | | | | |
| ***D*** × *D –* ***D*** × *M* | 1.3260 | 0.3871 | 3.425 | **0.0025** |
| *D* × ***D*** *–* *M* × ***D*** | -0.4466 | 0.2410 | -1.853 | 0.1277 |
| *D* × *D –* *M* × *M* | -0.4664 | 0.2263 | -2.061 | 0.1180 |
| *D* × *M –* *M* × *D* | -1.7726 | 0.3430 | -5.168 | **<0.0001** |
| *D* × ***M*** *–* *M* × ***M*** | -1.7924 | 0.3329 | -5.385 | **<0.0001** |
| ***M*** × *D –* ***M*** × *M* | -0.0198 | 0.1378 | -0.143 | 0.8860 |

m = *M*-race

Significance of the fixed factors included in the generalised linear model testing the number of eggs hatched per batch by each mating combination between host races of *H. diekei*. Significant P-values (α = 0.05) are highlighted in bold.

| Comparison | Factor | d.f. | Wald χ^2^ | P |
| --- | --- | --- | --- | --- |
| Factors determining the number of eggs hatched per batch | | | | |
|  | Male | 1 | 70.8178 | **<0.0001** |
|  | Female | 1 | 3.2222 | 0.0727 |
|  | Male × Female | 1 | 15.2623 | **<0.0001** |

**Table S8.** Results of the generalised linear model (GLM) testing hatching duration per egg batch from each mating combination between host races of *Henosepilachna diekei*. Significant P-values (α = 0.05) are highlighted in bold.

| Comparison | Estimate | SE | z value | Pr (> \| z \|) |
| --- | --- | --- | --- | --- |
| Factors determining the hatching duration | | | | |
| Intercept | 2.3979 | 0.0609 | 39.354 | **<0.0001** |
| Female (m) | 0.0494 | 0.0695 | 0.711 | 0.4774 |
| Male (m) | 0.2260 | 0.0874 | 2.587 | **0.0097** |
| Female (m) × Male (m) | -0.3106 | 0.0964 | -3.223 | **0.0013** |
| Pairwise comparison | | | | |
| ***D*** × *D –* ***D*** × *M* | -0.2260 | 0.0874 | -2.587 | **0.0484** |
| *D* × ***D*** *–* *M* × ***D*** | -0.0494 | 0.0695 | -0.711 | 0.9548 |
| *D* × *D –* *M* × *M* | 0.0352 | 0.0652 | 0.539 | 0.9548 |
| *D* × *M –* *M* × *D* | 0.1766 | 0.0710 | 2.489 | 0.0513 |
| *D* × ***M*** *–* *M* × ***M*** | 0.2612 | 0.0667 | 3.913 | **0.0005** |
| ***M*** × *D –* ***M*** × *M* | 0.0845 | 0.0407 | 2.077 | 0.1133 |

m = *M*-race

Significance of the fixed factors included in the generalised linear model testing hatching duration per egg batch from each mating combination between host races of *H. diekei*. Significant P-values (α = 0.05) are highlighted in bold.

| Comparison | Factor | d.f. | Wald χ^2^ | P |
| --- | --- | --- | --- | --- |
| Factors determining the hatching duration | | | | |
|  | Male | 1 | 5.4313 | **0.0198** |
|  | Female | 1 | 0.6244 | 0.4294 |
|  | Male × Female | 1 | 10.3857 | **0.0013** |

**Table S9.** Results of the generalised linear model (GLM) testing larval performance of parental and hybrid types of host races of *Henosepilachna diekei* across hosts and developmental stages (1). Significant P-values (α = 0.05) are highlighted in bold.

| Comparison | Estimate | SE | z value | Pr (> \| z \|) |
| --- | --- | --- | --- | --- |
| Factors determining the performance of the F_1_ larvae | | | | |
| Intercept | 1.5378 | 0.2260 | 6.806 | **<0.0001** |
| Female (*M*) | -0.8249 | 0.2917 | -2.828 | **0.0047** |
| Male (*M*) | -1.5789 | 0.5098 | -3.097 | **0.0019** |
| Food (*M*) | -5.7395 | 0.5423 | -10.584 | **<0.0001** |
| Female (*M*) × Male (*M*) | -3.8520 | 0.8119 | -4.745 | **<0.0001** |
| Female (*M*) × Food (*M*) | 6.0245 | 0.5721 | 10.530 | **<0.0001** |
| Male (*M*) × Food (*M*) | 5.4961 | 0.7577 | 7.254 | **<0.0001** |
| Female (*M*) × Male (*M*) × Food (*M*) | 2.0545 | 1.0135 | 2.027 | **0.0427** |

*M* = *M*-race (in case of sex) or *Mikania micrantha* (in case of food)

Significance of the fixed factors included in the generalised linear model testing larval performance of parental and hybrid types of host races of *Henosepilachna diekei* across hosts and developmental stages. Significant P-values (α = 0.05) are highlighted in bold.

| Comparison | Factor | d.f. | Wald χ^2^ | P |
| --- | --- | --- | --- | --- |
| Factors determining the performance of F_1_ larvae | | | | |
|  | Female | 1 | 0.6209 | 0.4307 |
|  | Male | 1 | 1.5674 | 0.2106 |
|  | Food | 1 | 0.8182 | 0.3657 |
|  | Female × Male | 1 | 20.2240 | **<0.0001** |
|  | Female × Food | 1 | 194.9226 | **<0.0001** |
|  | Male × Food | 1 | 167.4571 | **<0.0001** |
|  | Female × Male × Food | 1 | 4.1091 | **0.0427** |

**Table S10.** Results of the generalised linear model (GLM) testing larval performance of parental and hybrid types of host races of *Henosepilachna diekei* across hosts and developmental stages (2). Significant P-values (α = 0.05) are highlighted in bold.

| Comparison | Estimate | SE | t value | Pr (> \| z \|) |
| --- | --- | --- | --- | --- |
| Pairwise comparison (acceptance on *M. micrantha*) | | | | |
| ***D*** × *D –* ***D*** × *M* | -4.0690 | 0.9209 | -4.419 | **<0.0001** |
| *D* × ***D*** *–* *M* × ***D*** | -4.9885 | 0.7231 | -6.899 | **<0.0001** |
| *D* × *D –* *M* × *M* | -7.8482 | 1.0418 | -7.533 | **<0.0001** |
| *D* × *M –* *M* × *D* | -0.9195 | 0.6597 | -1.394 | 0.1634 |
| *D* × ***M*** *–* *M* × ***M*** | -3.7791 | 0.9988 | -3.487 | **0.0005** |
| ***M*** × *D –* ***M*** × *M* | -2.8597 | 0.8200 | -3.487 | **0.0009** |
| Pairwise comparison (survival to the 2^nd^ instar on *M. micrantha*) | | | | |
| ***D*** × *D –* ***D*** × *M* | -2.2773 | 1.2804 | -1.779 | 0.1010 |
| *D* × ***D*** *–* *M* × ***D*** | -4.1958 | 0.8948 | -4.689 | **<0.0001** |
| *D* × *D –* *M* × *M* | -7.1397 | 1.1108 | -6.427 | **<0.0001** |
| *D* × *M –* *M* × *D* | -1.9185 | 0.9810 | -1.956 | 0.1010 |
| *D* × ***M*** *–* *M* × ***M*** | -4.8624 | 1.1814 | -4.116 | **0.0002** |
| ***M*** × *D –* ***M*** × *M* | -2.9439 | 0.7463 | -3.945 | **0.0002** |
| Pairwise comparison (reaching adulthood on *M. micrantha*) | | | | |
| ***D*** × *D –* ***D*** × *M* | -2.6348 | 1.0981 | -2.399 | **0.0164** |
| *D* × ***D*** *–* *M* × ***D*** | -4.7958 | 0.8412 | -5.701 | **<0.0001** |
| *D* × *D –* *M* × *M* | -6.9561 | 0.8847 | -7.863 | **<0.0001** |
| *D* × *M –* *M* × *D* | -2.1609 | 0.7446 | -2.902 | **0.0074** |
| *D* × ***M*** *–* *M* × ***M*** | -4.3213 | 0.7934 | -5.447 | **<0.0001** |
| ***M*** × *D –* ***M*** × *M* | -2.1603 | 0.3622 | -5.965 | **<0.0001** |
| Pairwise comparison (acceptance on *D. canescens*) | | | | |
| ***D*** × *D –* ***D*** × *M* | 1.5782 | 0.8379 | 1.884 | 0.1706 |
| *D* × ***D*** *–* *M* × ***D*** | 0.9316 | 0.4892 | 1.904 | 0.1706 |
| *D* × *D –* *M* × *M* | 6.8024 | 1.1808 | 5.761 | **<0.0001** |
| *D* × *M –* *M* × *D* | -0.6466 | 0.7773 | -0.832 | 0.4055 |
| *D* × ***M*** *–* *M* × ***M*** | 5.2242 | 1.3264 | 3.939 | **0.0003** |
| ***M*** × *D –* ***M*** × *M* | 5.8708 | 1.1387 | 5.156 | **<0.0001** |
| Pairwise comparison (survival to the 2^nd^ instar on *D. canescens*) | | | | |
| ***D*** × *D –* ***D*** × *M* | 2.2336 | 0.9698 | 2.303 | 0.0558 |
| *D* × ***D*** *–* *M* × ***D*** | 1.1044 | 0.4492 | 2.459 | 0.0558 |
| *D* × *D –* *M* × *M* | 5.9915 | 1.3588 | 4.410 | **<0.0001** |
| *D* × *M –* *M* × *D* | -1.1291 | 0.9366 | -1.206 | 0.2280 |
| *D* × ***M*** *–* *M* × ***M*** | 3.7579 | 1.5880 | 1.366 | 0.0558 |
| ***M*** × *D –* ***M*** × *M* | 4.8870 | 1.3352 | 3.660 | **0.0013** |
| Pairwise comparison (reaching adulthood on *D. canescens*) | | | | |
| ***D*** × *D –* ***D*** × *M* | 2.0996 | 0.6562 | 3.200 | **0.0028** |
| *D* × ***D*** *–* *M* × ***D*** | 1.0010 | 0.2734 | 3.662 | **0.0010** |
| *D* × *D –* *M* × *M* | 5.6061 | 0.8800 | 6.371 | **<0.0001** |
| *D* × *M –* *M* × *D* | -1.0986 | 0.6431 | -1.708 | 0.0876 |
| *D* × ***M*** *–* *M* × ***M*** | 3.5066 | 1.0551 | 3.323 | **0.0027** |
| ***M*** × *D –* ***M*** × *M* | 4.6052 | 0.8703 | 5.292 | **<0.0001** |

**Table S11.** Results of the generalised linear model (GLM) testing larval performance of parental and hybrid types of host races of *Henosepilachna diekei* across hosts and developmental stages (3). Significant P-values (α = 0.05) are highlighted in bold.

| Comparison | Estimate | SE | t value | Pr (> \| t \|) |
| --- | --- | --- | --- | --- |
| Pairwise comparison (acceptance on different hosts) | | | | |
| Intercept | 2.1972 | 0.2934 | 7.488 | **<0.0001** |
| *D* × *D* on *M –* *D* × *D* on *D* | -5.8608 | 0.6356 | -9.221 | **<0.0001** |
| Intercept | 0.6190 | 0.7922 | 0.781 | 0.4530 |
| *D* × *M* on *M –* *D* × *M* on *D* | -0.2136 | 1.1056 | -0.193 | 0.8510 |
| Intercept | 1.2657 | 0.3329 | 3.802 | **0.0005** |
| *M* × *D* on *M –* *M* × *D* on *D* | 0.0593 | 0.4749 | 0.125 | 0.9013 |
| Intercept | -4.6050 | 0.7930 | -5.807 | **<0.0001** |
| *M* × *M* on *M –* *M* × *M* on *D* | 8.7900 | 1.0250 | 8.577 | **<0.0001** |
| Pairwise comparison (survival to the 2^nd^ instar on different hosts) | | | | |
| Intercept | 1.3863 | 0.2801 | 4.949 | **<0.0001** |
| *D* × *D* on *M –* *D* × *D* on *D* | -5.0499 | 0.7703 | -6.556 | **<0.0001** |
| Intercept | -0.8473 | 0.8103 | -1.046 | 0.355 |
| *D* × *M* on *M –* *D* × *M* on *D* | -0.5390 | 1.2323 | -0.437 | 0.684 |
| Intercept | 0.2819 | 0.3243 | 0.869 | 0.395 |
| *M* × *D* on *M –* *M* × *D* on *D* | 0.2504 | 0.4646 | 0.539 | 0.596 |
| Intercept | -4.605 | 1.1200 | -4.112 | **0.0007** |
| *M* × *M* on *M –* *M* × *M* on *D* | 8.081 | 1.2970 | 6.233 | **<0.0001** |
| Pairwise comparison (reaching adulthood on different hosts) | | | | |
| Intercept | 1.0010 | 0.1763 | 5.677 | **<0.0001** |
| *D* × *D* on *M –* *D* × *D* on *D* | -5.3704 | 0.7253 | -7.404 | **<0.0001** |
| Intercept | -1.0986 | 0.4808 | -2.285 | **0.0454** |
| *D* × *M* on *M –* *D* × *M* on *D* | -0.6360 | 0.7557 | -0.842 | 0.4197 |
| Intercept | 4.491×10^-17^ | 0.2103 | 0.000 | 1.0000 |
| *M* × *D* on *M –* *M* × *D* on *D* | 0.463 | 0.3009 | 1.417 | 0.1640 |
| Intercept | -4.6052 | 0.7689 | -5.989 | **<0.0001** |
| *M* × *M* on *M –* *M* × *M* on *D* | 7.1919 | 0.8253 | 8.714 | **<0.0001** |

**Table S12.** Results of the generalised linear model (GLM) testing host-associated assortative mating in host races of *Henosepilachna diekei*. Significant P-values (α = 0.05) are highlighted in bold.

| Comparison | Estimate | SE | z value | Pr (> \| z \|) |
| --- | --- | --- | --- | --- |
| Factors determining assortative mating | | | | |
| Intercept | 0.6931 | 0.5477 | 1.266 | 0.2057 |
| Male (m) | -0.2877 | 0.7601 | -0.378 | 0.7051 |
| Host (pres) | 2.0794 | 1.1673 | 1.781 | 0.0748 |
| Male (m) × Host (pres) | -0.3448 | 1.4829 | -0.233 | 0.8161 |
| Factors determining assortative mating in different spatial setting | | | | |
| Intercept | 2.9330 | 1.4102 | 2.080 | **0.0375** |
| Male (m) | -0.6603 | 1.2838 | -0.514 | 0.6070 |
| Arrangement (sep) | -0.2211 | 1.2916 | -0.171 | 0.8641 |

m = *M*-race; pres *=* present; sep *=* separate

Significance of the fixed factors included in the generalised linear model testing host-associated assortative mating in host races of *H. diekei*. Significant P-values (α = 0.05) are highlighted in bold.

| Comparison | Factor | d.f. | Wald χ^2^ | P |
| --- | --- | --- | --- | --- |
| Factors determining mating inferences in the absence/presence of host | | | | |
|  | Male | 1 | 0.3359 | 0.5622 |
|  | Host | 1 | 6.7161 | **0.0096** |
|  | Male × Host | 1 | 0.0541 | 0.8162 |
| Factors determining mating inferences in different spatial setting | | | | |
|  | Male | 1 | 0.2645 | 0.6070 |
|  | Arrangement | 1 | 0.0293 | 0.8641 |

**Table S13.** Details for migration and host fidelity data of host races of *Henosepilachna diekei* in field cages.

| Host races | No. collected adults | Sightings (*M/D/*N)^1)^ | Dispersal distance ± SE  (mean m day^-1^) | Intra-host migration frequency | Inter-host migration frequency |
| --- | --- | --- | --- | --- | --- |
| S - cage |  |  |  |  |  |
| *M ♀* | 14 | 100/ 0/ 9 | 0.442 ± 0.078 | 0.903 | 0 |
| *M ♂* | 12 | 76/ 1/ 13 | 0.726 ± 0.135 | 0.824 | 0 |
| *D ♀* | 10 | 2/ 80/ 5 | 0.757 ± 0.143 | 0.918 | 0.014 |
| *D ♂* | 12 | 2/ 75/ 8 | 0.747 ± 0.139 | 0.861 | 0.028 |
| C - cage |  |  |  |  |  |
| *M ♀* | 12 | 59/ 4/ 14 | 1.909 ± 0.248 | 0.714 | 0.079 |
| *M ♂* | 10 | 42/ 2/ 12 | 1.637 ± 0.282 | 0.690 | 0.024 |
| *D ♀* | 10 | 1/ 59/ 4 | 1.289 ± 0.274 | 0.901 | 0.019 |
| *D ♂* | 14 | 3/ 85/ 3 | 1.516 ± 0.177 | 0.908 | 0.053 |

^1)^*M =* on *Mikania micrantha*, *D =* on *Dicliptera canescens*, N = on net

**Table S14.** Results of the generalised linear model (GLM) testing host fidelity and dispersal of host races of *Henosepilachna diekei* in the field cage experiments. Significant P-values (α = 0.05) are highlighted in bold.

| Comparison | Estimate | SE | z value | Pr (> \| z \|) |
| --- | --- | --- | --- | --- |
| Factors determining the occurrence of beetle in the field cage | | | | |
| Intercept | -3.9807 | 0.7629 | -5.218 | **<0.0001** |
| Race (m) | 6.8292 | 0.9322 | 7.326 | **<0.0001** |
| Sex (male) | 0.1671 | 0.7772 | 0.215 | 0.8300 |
| Arrangement (sep) | 0.2393 | 0.7771 | 0.308 | 0.7580 |
| Race (m) × Sex (male) | -0.2273 | 1.1035 | -0.206 | 0.8370 |
| Race (m) × Arr. (sep) | 2.1093 | 1.3365 | 1.578 | 0.1150 |
| Factors determining the direction of migration in the field cage | | | | |
| Intercept | -5.0842 | 0.9217 | -5.516 | **<0.0001** |
| Race (m) | 7.6689 | 0.9273 | 8.270 | **<0.0001** |
| Sex (male) | 0.6842 | 0.8804 | 0.777 | 0.4370 |
| Arrangement (sep) | 1.4270 | 0.6817 | 2.093 | **0.0363** |
| Race (m) × Sex (male) | 0.5128 | 1.4162 | 0.362 | 0.7173 |
| Factors determining the dispersal distance in the field cage | | | | |
| Intercept | 0.2535 | 0.1622 | 1.563 | 0.1191 |
| Race (m) | 0.3928 | 0.2087 | 1.882 | 0.0607 |
| Sex (male) | 0.1627 | 0.2042 | 0.797 | 0.4262 |
| Arrangement (sep) | -0.5320 | 0.2497 | -2.130 | **0.0339** |
| Race (m) × Sex (male) | -0.3162 | 0.2957 | -1.069 | 0.2857 |
| Race (m) × Arr. (sep) | -0.9315 | 0.3491 | -2.669 | **0.0080** |
| Sex (male) × Arr. (sep) | -0.1753 | 0.3380 | -0.519 | 0.6045 |
| Race (m) × Sex (male) × Arr. (sep) | 0.8251 | 0.4871 | 1.694 | 0.0913 |

mrace = *M*-race; sep *=* separate; Arr = arrangement

Significance of the fixed factors included in the generalised linear model testing host fidelity and dispersal of host races of *H. diekei* in the field cage experiments. Significant P-values (α = 0.05) are highlighted in bold.

| Comparison | Factor | d.f. | Wald χ^2^ | P |
| --- | --- | --- | --- | --- |
| Factors determining the occurrence of beetle in the field cage | | | | |
|  | Race | 1 | 143.2514 | **<0.0001** |
|  | Sex | 1 | 0.0097 | 0.9215 |
|  | Arrangement | 1 | 2.2691 | 0.1320 |
|  | Race × Sex | 1 | 0.0424 | 0.8368 |
|  | Race × Arrangement | 1 | 2.4907 | 0.1145 |
| Factors determining the direction of migration in the field cage | | | | |
|  | Race | 1 | 106.6109 | **<0.0001** |
|  | Sex | 1 | 1.6408 | 0.2002 |
|  | Arrangement | 1 | 4.3817 | **0.0363** |
|  | Race × Sex | 1 | 0.1311 | 0.7173 |
| Factors determining the dispersal distance in the field cage | | | | |
|  | Race | 1 | 0.1661 | 0.6836 |
|  | Sex | 1 | 0.6364 | 0.4250 |
|  | Arrangement | 1 | 52.3072 | **<0.0001** |
|  | Race × Sex | 1 | 0.0027 | 0.9587 |
|  | Race × Arrangement | 1 | 4.3506 | **0.0370** |
|  | Sex × Arrangement | 1 | 0.8326 | 0.3615 |
|  | Race × Sex × Arr. | 1 | 2.8695 | 0.0903 |

**Table S15.** Empirical reproductive isolation (*RI*) estimates classified according to the reproductive isolation ontology (RIO) framework.

| Lineage I | Lineage II | RI barrier | RIO codes (lineage I) | RIO codes (lineage II) |
| --- | --- | --- | --- | --- |
| Without host-plants (laboratory) | | | | |
| *H. diekei* (*M*-race) | *H. diekei* (*D*-race) | habitat | 1 [C0010; D0039; T0007] | 1 [C0010; D0043; T0010] |
|  |  | sexual | 0.6364 [C0040; D0003; T0007] | 0.2 [C0040; D0007; T0010] |
|  |  | prehatching | 0.002 [C0000; D0048; T0007] | 0.4888 [C0000; D0052; T0010] |
|  |  | hybrid inviability | 0.2109 [C0005; D0048; T0007] | 0.5362 [C0005; D0052; T0010] |
| *H. diekei* (*M*-race) | *H. diekei* (*L*-race) | habitat | 1 [C0010; D0039; T0009] | 1 [C0010; D0043; T0010] |
|  |  | sexual | -0.0803 [C0040; D0003; T0009] | 0.0194 [C0040; D0007; T0010] |
|  |  | prehatching | 0.0147 [C0000; D0048; T0009] | -0.0265 [C0000; D0052; T0010] |
|  |  | hybrid inviability | -0.0078 [C0005; D0048; T0009] | 0.0125 [C0005; D0052; T0010] |
| With host-plants (field-cage) | | | | |
| *H. diekei* (*M*-race) | *H. diekei* (*D*-race) | habitat (sympatry-like) | 0.8879 [C0010; D0039; T0007] | 0.9459 [C0010; D0043; T0011] |
|  |  | habitat (parapatry-like) | 0.9887 [C0010; D0039; T0007] | 0.9497 [C0010; D0043; T0011] |
|  |  | sexual (sympatry-like) | 0.75 [C0040; D0003; T0007] | 1 [C0040; D0007; T0011] |
|  |  | sexual (parapatry-like) | 0.8182 [C0040; D0003; T0007] | 0.8333 [C0040; D0007; T0011] |
| *H. diekei* (*M*-race) | *H. diekei* (*L*-race) | habitat (sympatry-like) | 0.9901 [C0010; D0039; T0009] | 1 [C0010; D0043; T0013] |
|  |  | habitat (parapatry-like) | 0.9301 [C0010; D0039; T0009] | 0.9815 [C0010; D0043; T0013] |
|  |  | sexual (sympatry-like) | 1 [C0040; D0003; T0009] | 1 [C0040; D0007; T0013] |
|  |  | sexual (parapatry-like) | 0.8824 [C0040; D0003; T0009] | 0.7647 [C0040; D0007; T0013] |
